# GTP Hydrolysis of TC10 Promotes Neurite Outgrowth through Exocytic Fusion of Rab11- and L1-Containing Vesicles by Releasing Exocyst Component Exo70

**DOI:** 10.1371/journal.pone.0079689

**Published:** 2013-11-04

**Authors:** Akane Fujita, Shingo Koinuma, Sayaka Yasuda, Hiroyuki Nagai, Hiroyuki Kamiguchi, Naoyuki Wada, Takeshi Nakamura

**Affiliations:** 1 Division of Biosignaling, Research Institute for Biomedical Sciences, Tokyo University of Science, Noda, Chiba, Japan; 2 Laboratory for Neuronal Growth Mechanism, RIKEN Brain Science Institute, Wako, Saitama, Japan; 3 Department of Applied Biological Science, Tokyo University of Science, Noda, Chiba, Japan; UPR 3212 CNRS -Université de Strasbourg, France

## Abstract

The use of exocytosis for membrane expansion at nerve growth cones is critical for neurite outgrowth. TC10 is a Rho family GTPase that is essential for specific types of vesicular trafficking to the plasma membrane. Recent studies have shown that TC10 and its effector Exo70, a component of the exocyst tethering complex, contribute to neurite outgrowth. However, the molecular mechanisms of the neuritogenesis-promoting functions of TC10 remain to be established. Here, we propose that GTP hydrolysis of vesicular TC10 near the plasma membrane promotes neurite outgrowth by accelerating vesicle fusion by releasing Exo70. Using Förster resonance energy transfer (FRET)-based biosensors, we show that TC10 activity at the plasma membrane decreased at extending growth cones in hippocampal neurons and nerve growth factor (NGF)-treated PC12 cells. In neuronal cells, TC10 activity at vesicles was higher than its activity at the plasma membrane, and TC10-positive vesicles were found to fuse to the plasma membrane in NGF-treated PC12 cells. Therefore, activity of TC10 at vesicles is presumed to be inactivated near the plasma membrane during neuronal exocytosis. Our model is supported by functional evidence that constitutively active TC10 could not rescue decrease in NGF-induced neurite outgrowth induced by TC10 depletion. Furthermore, TC10 knockdown experiments and colocalization analyses confirmed the involvement of Exo70 in TC10-mediated trafficking in neuronal cells. TC10 frequently resided on vesicles containing Rab11, which is a key regulator of recycling pathways and implicated in neurite outgrowth. In growth cones, most of the vesicles containing the cell adhesion molecule L1 had TC10. Exocytosis of Rab11- and L1-positive vesicles may play a central role in TC10-mediated neurite outgrowth. The combination of this study and our previous work on the role of TC10 in EGF-induced exocytosis in HeLa cells suggests that the signaling machinery containing TC10 proposed here may be broadly used for exocytosis.

## Introduction

Vast surface expansion of neurons during the formation of axons and dendrites necessitates polarized transport of membrane and membrane proteins primarily at growth cones [1]. Exocytosis for membrane expansion is different from that for the release of synaptic vesicles [2,3]. The recruitment of membrane and membrane proteins for outgrowth is realized by exocytosis of plasmalemmal precursor vesicles (PPVs) at growth cones [1,4,5]. In growth cones, the transitional zone between microtubule-rich central domain and actin-rich peripheral domain frequently contains PPVs, which are large, clear, and coat-free vesicles clustered against the plasma membrane [4,6]. *Plasmalemmal expansion* in neurons consists of targeting of specific vesicle types to axons versus dendrites, long-vesicle transport, and growth factor-regulated mechanisms of vesicle insertion at growth tips. Neurite outgrowth requires the regulation of membrane trafficking and cytoskeletal reorganization, and coordination between these processes is critical. The understanding of signaling pathways from extracellular stimulation to the mechanisms regulating cytoskeletal reorganization in neurons has remarkably advanced in the past two decades [7,8]. By contrast, the machineries that coordinate complicated trafficking pathways during neurite outgrowth are yet to be fully elucidated, and thus the mechanisms linking membrane trafficking and cytoskeletal reorganization are an important subject to be resolved.

Recent studies have highlighted the role of TC10, a Rho family GTPase, in membrane trafficking and neurite outgrowth [9-11]. TC10 is localized to vesicular structures and the plasma membrane [12]. It has been shown that TC10 plays a significant role in the exocytosis of GLUT4 [13,14] and other proteins by tethering their vesicles to the plasma membrane [15,16]. Exo70 is a component of the exocyst tethering complex [17] and an effector of TC10 [18]. Recent studies have indicated that a TC10-Exo70 complex is essential for membrane expansion in developing hippocampal neurons and axon regeneration [10,11]. These studies have argued that activation of plasmalemmal TC10 triggers translocation of Exo70 toward the plasma membrane in growth cones [19]. However, this argument lacks direct evidences because of technical difficulties. Thus, others have raised objections from two points of view. One point at issue is the regulation of TC10 activity at, and in the close proximity to, the plasma membrane following stimulation of exocytosis. Using a Förster resonance energy transfer (FRET) sensor for TC10, we previously found that TC10 activity at tethered vesicles dropped immediately before vesicle fusion in EGF-stimulated HeLa cells [20]. Another point of debate is the primary location of Exo70. Recent study in HeLa cells has indicated that a considerable amount of Exo70 is localized to peripheral and perinuclear endosomes [21], while several studies reported that Exo70 associates with the plasma membrane through interacting with PI(4,5)P_2_ [22,23].

In the present study, we have provided evidence for a mechanistic model in which the neuritogenesis-promoting functions of TC10 are executed by triggering vesicle fusion through release of Exo70 following its GTP hydrolysis. TC10 activity at the plasma membrane was lower at extending growth cones. In neuronal cells, TC10 activity on vesicles was higher than that at the plasma membrane and TC10-positive vesicles were found to fuse to the plasma membrane. Exo70 was clearly localized to vesicular structures in growth cones and neurite shafts, and active TC10 on vesicles was bound to its effectors including Exo70. Thus, the putative TC10 inactivation on tethered vesicles in neuronal cells can induce Exo70 release and exocyst disassembly, which contributes to vesicle fusion. Our model has been functionally supported by the finding that a GTPase-deficient TC10 mutant could not rescue the decrease in nerve growth factor (NGF)-induced neurite outgrowth following TC10 depletion. Furthermore, the characterization of TC10-containing vesicles suggests that exocytic fusion of Rab11- and L1-containing PPVs plays a central role in the promoting effects of TC10 in neuritogenesis. This study has extended our previous work on the role of TC10 in EGF-induced exocytosis in HeLa cells to clarify the mechanisms underlying TC10’s functions in neurite outgrowth. The signaling machinery proposed here may be broadly used for exocytosis.

## Materials and Methods

### Ethics Statement

All animal procedures were done using approved protocols by the Tokyo University of Science Animal Care and Use Committee (Permit Number: S12040). All surgeries were performed under anesthesia, and all efforts were made to minimize suffering.

### FRET Biosensors

The plasmids we used to encode the FRET biosensors, Raichu-TC10/TC10-CT (1096x) and Raichu-TC10/K-RasCT (1096kx), have been described previously [20]. Raichu-TC10/TC10-CT contained the carboxyl-terminal region of TC10 (TC10-CT) in its C-terminus and the distribution of Raichu-TC10/TC10-CT was indistinguishable from that of authentic TC10. Thus, we use Raichu-TC10/TC10-CT to examine the difference in activity between vesicular TC10 and plasmalemmal one. In Raichu-TC10/K-RasCT, the TC10-CT in Raichu-TC10/TC10-CT was replaced with the carboxyl-terminal region of K-Ras and thereby Raichu-TC10/K-RasCT was localized to the plasma membrane. An excess of cytosolic sensors that bind to RhoGDI profoundly decrease the signal-to-noise ratio. The use of Raichu-TC10/K-RasCT can circumvent this difficulty. As far as the plasma membrane is concerned, these two FRET sensors provide comparable information for TC10 activity as previously described [20].

### Plasmids

The RNA targeting constructs were generated using a pRFP-C-RS vector (OriGene, Rockville, MD, USA). The 29-nucleotide sequences used to target rat TC10 mRNA was 5′-CTGTCTTCGACCACTACGCAGTCAGCGTC-3′. The full-length human TC10 cDNA [20] was point-mutated to resist short-hairpin RNA (shRNA)-mediated knockdown and was subcloned into the pCAGGS-FLAG vector [24] to generate pCAGGS-FLAG-resi-TC10. The cDNAs for human TC10 and dog Rab11a were subcloned into pCAGGS-mCherry. The cDNA for human TC10 was subcloned into pCAGGS-mTFP and pCMV-FLAG to generate pCAGGS-mTFP-TC10 and pCMV-FLAG-TC10, respectively. The cDNA for rat Exo70 (a gift from Y. Ohta, Kitasato University, Tokyo, Japan) was subcloned into the pEGFP-N1 vector. pERedNLS-Rac1-S17N, pERedNLS-Rac1-G12V, pCS2-L1-Venus, pCS2-L1-GFP and pCS2-VAMP2-Venus have been described previously [25-27]. The cDNA for VAMP7-Venus (a gift from A. Miyawaki, RIKEN Brain Science Institute, Saitama, Japan) was used to prepare pCS2-VAMP7-Venus.

### Cells, Reagents, and Antibodies

PC12 cells [28] were maintained in Roswell Park Memorial Institute medium (Invitrogen, Carlsbad, CA, USA) supplemented with 10% horse serum and 5% fetal bovine serum (FBS). The cells were plated on 35-mm glass-base dishes (Asahi Techno Glass, Tokyo, Japan) coated with polyethyleneimine (Sigma-Aldrich, St. Louis, MO, USA). If necessary, the glass-base dishes were additionally coated with laminin (Invitrogen). PC12 cells respond well to extracellular stimulation such as NGF and dibutyryl cAMP (dbcAMP) with a good reproducibility. Thus we use the cells in the experiments using extracellular stimulations. N1E-115 mouse neuroblastoma cells [29] were kindly provided by H. Higashida (Kanazawa University Graduate School of Medicine, Ishikawa, Japan) and grown in Dulbecco’s modified Eagle’s medium (DMEM) containing 5% FBS. The cells were plated on 35-mm glass-base dishes coated with polyethyleneimine and laminin. In a serum-free (i.e., low level of LPA) condition, N1E-115 cells develop long neurites and their growth cone frequently extend widely. Thus we use N1E-115 cells to examine the events in growth cones as much as possible. Hippocampal neurons were prepared from E18 rat embryos according to a standard method [30] with a slight modification. Hippocampal neurons were dissociated with 0.25% trypsin and 1 mM EDTA at 37°C for 10 min, and then plated on glass-base dishes coated with polyethyleneimine in a neuronal plating medium (MEM containing 20% glucose, 1 mM pyruvic acid, and 10% FBS). After incubation for 3 h, the medium was replaced with conditioned medium from glia, which was prepared separately with phenol red-free MEM (Invitrogen) for FRET imaging. NGF and dbcAMP were purchased from Calbiochem (La Jolla, CA, USA). Apocynin was obtained from Tokyo Chemical Industry (Tokyo, Japan). Puromycin was obtained from Sigma-Aldrich. The following primary antibodies were used: rabbit monoclonal antibody to TC10 (abcam, Cambridge, UK); mouse monoclonal antibody to Exo70 (70X12F3, Merck Millipore, Billerica, MA, USA); mouse monoclonal antibodies to Rab11, β1-integrin and N-cadherin (BD Biosciences, San Jose, CA, USA); anti-FLAG-M2 mouse monoclonal antibody (Sigma-Aldrich); rabbit polyclonal antibodies to a DYKDDDDK peptide (Cell Signaling, Danvers, MA, USA); rabbit polyclonal antibodies to GAPDH (Santa Cruz Biotechnology, Santa Cruz, CA, USA); rat monoclonal antibody to HA (3F10; Roche Diagnostics, Indianapolis, IN, USA). Rabbit polyclonal antibodies against rat L1 [31] was kindly provided by V. Lemmon (University of Miami, Miami, FL, USA).

### RNA Interference Experiments

PC12 cells were transfected with the desired shRNA constructs using Lipofectamine 2000 (Invitrogen). After recovery, the cells were selected by a 2-d incubation with 2 μg/ml puromycin and then used for further analysis.

### Neurite Outgrowth Assay

PC12 cells were transfected with the indicated shRNA constructs and selected with 2 µg/ml puromycin for 2 d if necessary. Then, neurite outgrowth was induced with 50 ng/ml NGF and allowed to proceed for 48 h in DMEM/F-12 medium containing 0.1% bovine serum albumin (BSA) and 2 µg/ml puromycin. Quantification of neurite outgrowth was performed as described previously [32].

### Time-Lapse Imaging

PC12 cells expressing FRET biosensors were starved for 30 min with phenol red-free DMEM/F-12 medium (Invitrogen) containing 0.1% BSA and then treated with 50 ng/ml NGF. N1E-115 cells expressing FRET biosensors were serum-starved for 6 h before imaging. The medium was covered with mineral oil (Sigma-Aldrich) to preclude evaporation. Cells were imaged with an IX81 inverted microscope (Olympus, Tokyo, Japan) equipped with a Cool SNAP-HQ cooled charge-coupled device camera (Roper Scientific, Trenton, NJ, USA), a laser auto-focusing system (IX2-ZDC, Olympus) and an automatically programmable XY stage (MD-XY30100T-Meta, SIGMA KOKI, Tokyo, Japan), which allowed us to obtain time-lapse images of several fields of view in a single experiment. The following filters were used for the dual-emission imaging: an FF01-438/24-25 excitation filter (Semrock, Rochester, NY, USA); an XF2034 (455DRLP) dichroic mirror (Omega Optical, Brattleboro, VT, USA); and two emission filters (FF01-483/32-25 for CFP and FF01-542/27-25 for FRET, Semrock). Cells were illuminated with a 75-W Xenon lamp through a 6% neutral density filter and viewed through a 60× oil immersion objective lens (PlanApo 60×/1.4, Olympus). The exposure times for 4 × 4 binning were 200 ms for CFP and FRET images and 100 ms for differential interference contrast (DIC) images. After background subtraction, FRET/CFP ratio images were created with the MetaMorph software (Universal Imaging, West Chester, PA, USA), and the images were used to represent FRET efficiency. We compared the FRET/CFP ratios between extending and stationary regions in NGF-treated PC12 cells as follows: For each cell, we selected three ROIs (20 pixels × 20 pixels) in extending regions mainly in neurite tips and in stationary regions mainly in cell body and neurite shafts. We made the judgment for motility by sight.

### Confocal Microscopy

For fluorescence imaging, cells were fixed with 3.7 % formaldehyde and permeabilized with 0.2% Triton X-100. After being soaked in phosphate-buffered saline containing 3% BSA for 1 h, the samples were incubated overnight with a primary antibody, and then incubated for 1 h with fluorescent secondary antibody (conjugated to Alexa Fluor 488 or 594, Invitrogen). The samples were imaged with an IX81 inverted microscope (Olympus) equipped with an FV-1000 confocal imaging system (Olympus).

For confocal FRET imaging, PC12 or N1E-115 cells expressing Raichu-TC10/TC10-CT were cultured for 24 h in DMEM/ F-12 medium with 50 ng/ml NGF (PC12) or for 6 h in serum-free DMEMF-12 medium (N1E-115), respectively. The cells were then imaged with the IX81 inverted microscope equipped with the FV-1000 confocal imaging system. The excitation laser and fluorescence filter settings were as follows: excitation laser, 440 nm; excitation dichroic mirror, DM405-440; CFP channel PMT dichroic mirror, SDM 510; CFP channel PMT filter, BA460-490; FRET channel PMT filter, BA515-615. Image processing was performed with MetaMorph software.

### Time-lapse Total Internal Reflection Fluorescence (TIRF) Microscopy of Fusion of Exocytotic Vesicles

TIRF studies were conducted using an Olympus IX70 inverted microscope equipped with a 442 nm HeCd laser (Omnichrome, Chino, CA, USA), a TIRF illuminator, and a 100× oil immersion objective lens for TIRF microscopy (PlanApo 100×/1.4, Olympus). Images were obtained using an EMCCD camera (iXon DV887, Andor, Belfast, UK). Stacked images containing 1500 planes were continuously acquired with a 200 ms exposure. Image processing was performed with MetaMorph software.

Dual-color images of L1-GFP and mCherry-TC10 were obtained by simultaneous TIRF microscopy [33]. Both fluorescent proteins were excited with 488 nm (Melles Griot, Rochester, NY, USA) and 561 nm (LASOS Lasertechnik, Jena, Germany) solid-state lasers. The EGFP and mCherry emissions were split and acquired simultaneously with a CCD camera (ORCA D2, Hamamatsu Photonics, Hamamatsu, Japan).

### In Vitro Analysis of TC10 Activity

TC10 activity in dbcAMP-treated PC12 cells was measured by Bos’ pull-down method using GST-PAK-CRIB domain fusion protein as described previously [25]. Proteins bound to GST-PAK-CRIB and total lysates were analyzed by immunoblotting with an anti-HA antibody.

## Results

### TC10 activity at the plasma membrane is lower at extending growth cones in neuronal cells

**Figure 1:**
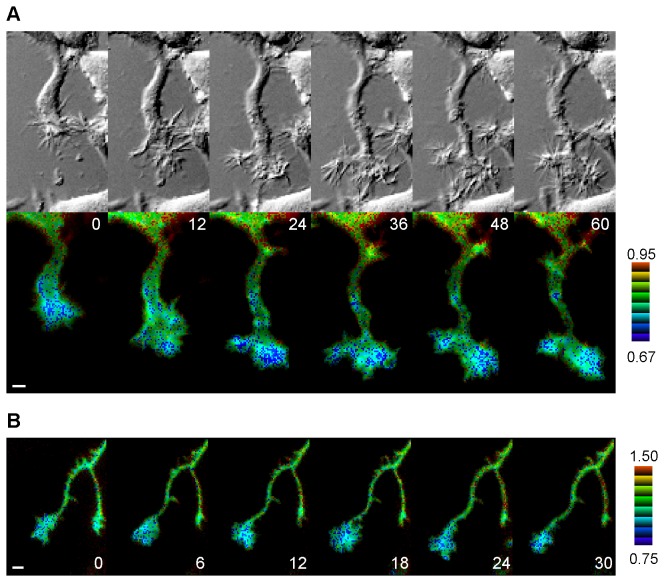
Distribution of TC10 activity at the plasma membrane of extending neurites. (***A***) PC12 cells expressing Raichu-TC10/K-RasCT were treated with 50 ng/ml NGF for 24 h and then imaged every 2 min for 1 h. Representative images of the ratio of FRET/CFP at the indicated time points (in min) are shown in an intensity-modulated display (IMD) mode with the corresponding DIC images. In the IMD mode, eight colors from red to blue are used to represent the FRET/CFP ratio, with the intensity of each color indicating the mean intensity of FRET and CFP. The upper and lower limits of the ratio images are shown on the right. A bar, 5 μm. (***B***) Hippocampal neurons expressing Raichu-TC10/K-RasCT were imaged every 3 min for 30 min. Representative ratio images of FRET/CFP at the indicated time points (in min) are shown as described for (***A***). A bar, 10 μm.

Using a FRET-based biosensor, we examined spatiotemporal changes in TC10 activity at the plasma membrane in PC12 cells, which extend neurites in the presence of NGF. Previous spectrofluorometric analyses and biochemical experiments showed that Raichu-TC10, a sensor for TC10 activity, can reliably monitor the balance between GEF and GAP activities acting on TC10 [20]. Figure 1A shows that TC10 was locally inactivated at protruding neurite tips in NGF-treated PC12 cells (Video S1). Extending regions showed a 14.4±2.8% decrease in the FRET/CFP ratio in comparison to stationary regions (n = 10). Down-regulation of TC10 occurred over a growth cone, although a peripheral area showed a clearer decrease in TC10 activity than the neck of the growth cone. It is worth noting that this result refutes the previous view, in which plasmalemmal TC10 may be activated at growth cones in hippocampal neurons [11]. This idea is based on indirect evidence collected by examining the amount of plasmalemmal Exo70 using TIRF microscopy. Thus, we performed FRET imaging of hippocampal neurons expressing Raichu-TC10/K-RasCT. As also shown in PC12 cells, extending growth cones in hippocampal neurons clearly showed lower TC10 activity (Figure 1B). The FRET/CFP ratio in spreading regions was 15.5±3.2% lower than that in static areas (n = 10).

### Rac1 and reactive oxygen species (ROS) mediate NGF-induced TC10 inactivation at the plasma membrane

**Figure 2:**
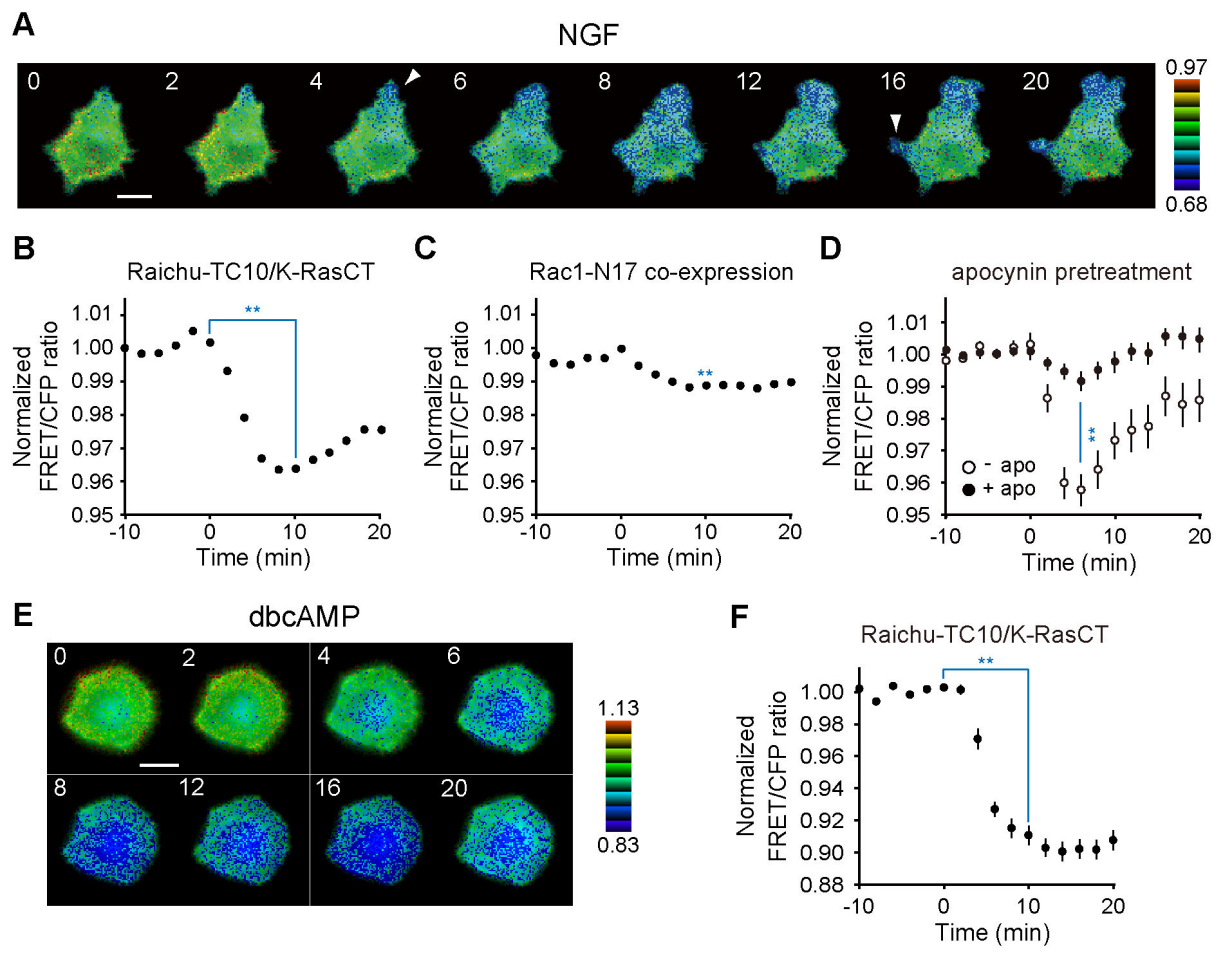
Spatiotemporal changes in TC10 activity at the plasma membrane following NGF or dbcAMP stimulation. (***A and B***) PC12 cells expressing Raichu-TC10/K-RasCT were starved for 30 min and then stimulated with 50 ng/ml NGF. Images were obtained every 2 min for 20 min after NGF addition. (***A***) Representative images of the ratio of FRET/CFP at the indicated time points (in min) after NGF stimulation are shown as described for Figure 1A. A bar, 10 μm. (***B***) The mean FRET/CFP ratios averaged over the whole cell were determined by measuring the relative increase compared with the reference value, which was averaged over 10 min before NGF stimulation. Error bars show the SE (n = 55). The blue symbol indicates the result of a Student’s *t* test analysis (**p < 0.01). (***C***) PC12 cells were cotransfected with pRaichu-TC10/K-RasCT and pERedNLS-Rac1-S17N, stimulated with NGF, and then imaged every 2 min. The mean FRET/CFP ratios averaged over the whole cell are expressed in the same manner as in the legend to Figure 2B. Error bars show the SE (n = 58). The blue symbol indicates a significant difference between the value at 10 min after NGF addition in the control cells (***B***) and the one in the Rac1-S17N-expressing cells in a Student’s *t* test analysis (**p < 0.01). (***D***) PC12 cells expressing Raichu-TC10/K-RasCT were treated with 10 μM apocynin for 30 min, stimulated with NGF, and then imaged every 2 min. The mean FRET/CFP ratios averaged over the whole cell are expressed in the same manner as in the legend to Figure 2B. Error bars show the SE. The number of experiments for the control condition was 14 and for the apocynin pretreatments was 24. The blue symbol indicates the result of a Student’s t test analysis (**p < 0.01). (***E and F***) PC12 cells expressing Raichu-TC10/K-RasCT were starved for 30 min and then treated with 1 mM dbcAMP. Images were obtained every 2 min for 20 min after dbcAMP addition. (***E***) Representative images of the ratio of FRET/CFP at the indicated time points (in min) after dbcAMP treatment are shown as described for Figure 1A. A bar, 10 μm. (***F***) The mean FRET/CFP ratios averaged over the whole cell are expressed in the same manner as in to legend to Figure 2B. Error bars show the SE (n = 30). The blue symbol indicates the result of a Student’s *t* test analysis (**p < 0.01).

To investigate the mechanisms underlying GTP hydrolysis of TC10 at extending neurite tips and growth cones, we examined plasmalemmal TC10 activity for 20 min after NGF stimulation in naïve PC12 cells expressing Raichu-TC10/K-RasCT. The FRET/CFP ratio dropped to its nadir within 10 min after NGF addition (Figure 2A and 2B; Video S2). *While lower* TC10 activity occurred throughout the cells, the protruding sites showed a prominent decrease in GTP-TC10 activity (arrowheads in Figure 2A). This corresponds to the specific inactivation of TC10 at the extending neurite tips and growth cones shown in Figure 1. In previous work, we showed that GTP hydrolysis of plasmalemmal TC10 in EGF-stimulated HeLa cells depended on active Rac and redox-regulated p190RhoGAP [20]. Because NGF-stimulated PC12 cells showed an initial global increase in GTP-Rac1 and subsequent local Rac1 activation at induced protrusions [25], we examined the effects of a dominant-negative mutant of Rac1, Rac1-S17N, on TC10 inactivation following NGF stimulation. We found that Rac1-S17N almost completely blocked NGF-induced decreases in TC10 activity in PC12 cells (Figure 2C). NGF-induced TC10 inactivation was slightly mitigated by the expression of constitutively active mutant of Rac1 (Figure S1A); the incomplete inhibition of TC10 inactivation was partly because cells could be adapted to the chronic perturbation by Rac1-G12V expression, although the precise mechanism is unknown. Rac1 activates p190RhoGAP via ROS [34] and p190RhoGAP stimulates the GTPase activity of TC10 [20]. Thus, we tested the effects of apocynin and diphenyleneiodonium chloride (DPI), inhibitors of ROS generation. Figures 2D and S1B showed that apocynin and DPI efficiently inhibited NGF-induced TC10 inactivation. These data suggested that, as in EGF-treated HeLa cells, active Rac1 and ROS caused NGF-induced GTP hydrolysis of TC10 at the plasma membrane, possibly through redox-regulated p190RhoGAP. As well as NGF, the second messenger cAMP strongly promotes neurite outgrowth [35]. Thus, we examined changes in plasmalemmal TC10 activity following dbcAMP treatment in PC12 cells. TC10 activity gradually decreased throughout the cells over 10 min after dbcAMP addition, and remained at its minimal level during the succeeding 10 min (Figures 2E and 2F) and later. TC10 inactivation following dbcAMP treatment was confirmed by pull-down experiments using a GST-PAK-CRIB domain fusion protein (Figure S1C).

### TC10 activity at vesicles is higher than that at the plasma membrane in neuronal cells

**Figure 3:**
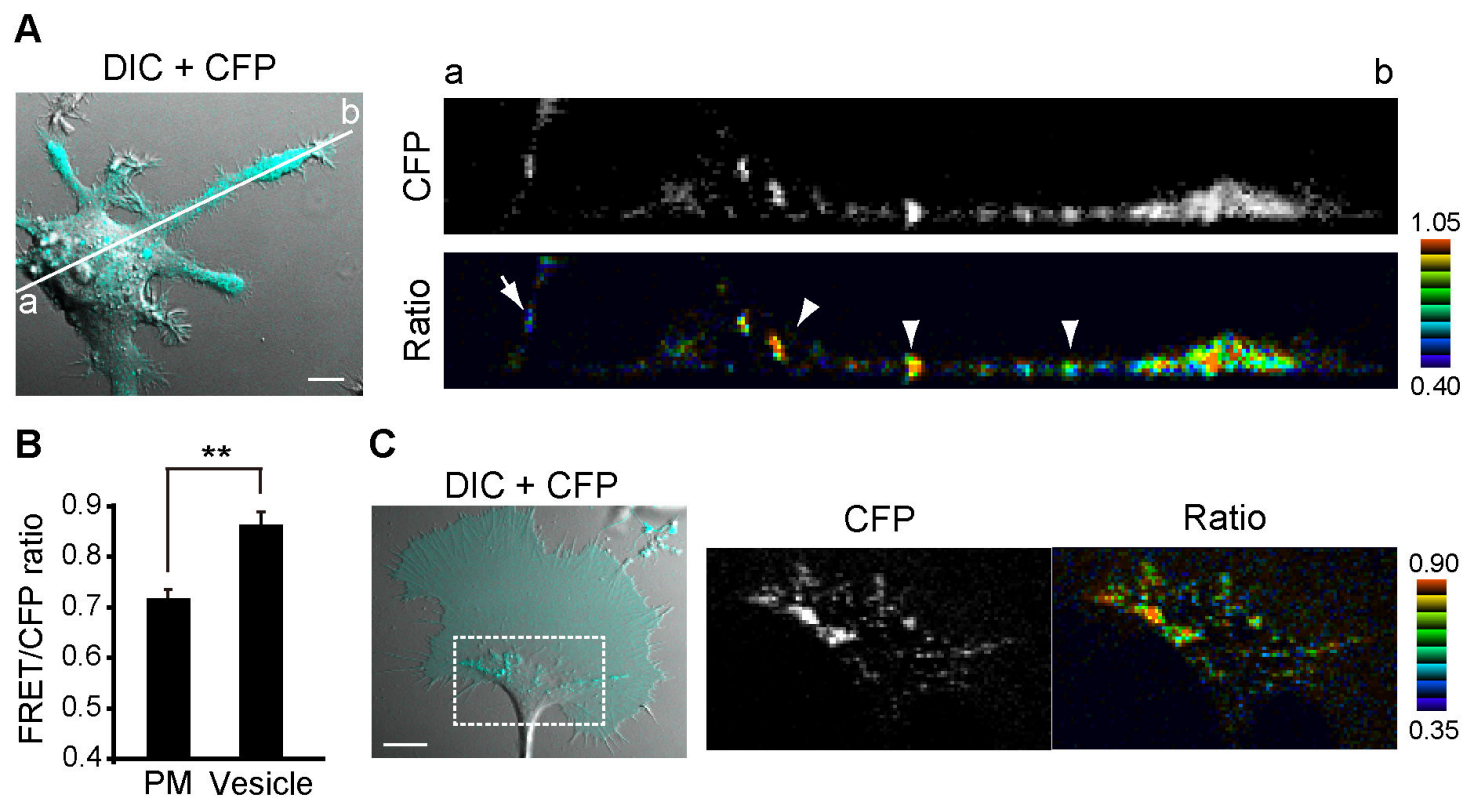
Distribution of TC10 activity in neuronal cells. (***A and B***) PC12 cells expressing Raichu-TC10/TC10-CT were cultured with NGF for 24 h and observed by confocal microscopy for FRET imaging. (***A***) The left panel shows a representative CFP image superimposed with a corresponding DIC image. In the bottom-right panel, one X-Z slice of the FRET/CFP ratio image corresponding to the white line in the left panel (from a to b) is shown. The ratio image is shown in the IMD mode. The upper and lower limits of the ratio image are shown on the right. The top right panel shows the corresponding CFP image. A bar, 10 μm. (***B***) A bar graph represents the average plus SE of the FRET/CFP ratio on the plasma membrane or vesicles. The symbol indicates a significant difference in a Student’s *t* test analysis (**p < 0.01). (***C***) N1E-115 cells expressing Raichu-TC10/TC10-CT were serum-starved for 6 h to promote growth cone spreading and examined by confocal FRET imaging. The left panel shows a CFP image of a growth cone superimposed with a corresponding DIC image. The right panel shows the CFP and FRET/CFP ratio images corresponding to the boxed region in the left panel. A bar, 10 μm.

Because TC10 is localized to vesicles and the plasma membrane [12], we examined the spatial distribution of the TC10 activity in NGF-treated PC12 cells using Raichu-TC10/TC10-CT by confocal microscopy. The FRET/CFP ratio at vesicles (arrowheads in Figure 3A, ratio image) was 19.4% (n = 10) higher than that at the plasma membrane (an arrow in Figure 3A, ratio image; Figure 3B). Similarly, in hippocampal neurons, the FRET/CFP ratio at vesicles was 20.2% (n = 10) higher than the plasmalemmal ratio (Figure S2). Some TC10-positive vesicles fused to the plasma membrane in EGF-treated HeLa cells [20]. Considering TC10 inactivation at the plasma membrane in extending growth cones (Figure 1) and TC10 activity at vesicles was higher than its plasmalemmal activity, TC10 activity at vesicles is presumed to drop during vesicle fusion in neuronal cells. Furthermore, we have noticed that TC10 on individual vesicles showed various levels of activity in growth cones (Figure 3C), although a functional meaning of the diversity is still unknown.

### Constitutively active TC10 cannot rescue the decrease in NGF-induced neurite outgrowth by TC10 depletion

**Figure 4:**
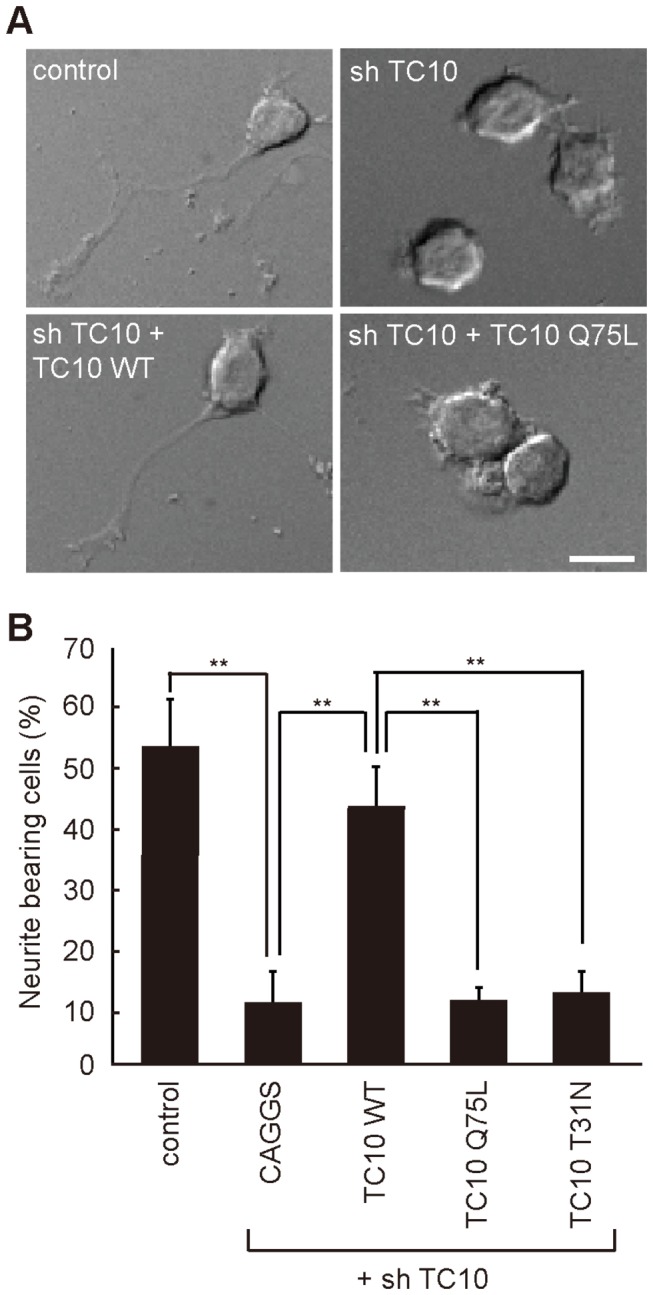
Effect of TC10 depletion on NGF-induced neurite outgrowth in PC12 cells. PC12 cells were transfected with pCAGGS-Flag-resi-TC10-WT or its mutants in combination with a TC10-targeted shRNA vector. After selection with puromycin, the selected cells were cultured with 50 ng/ml NGF for 2 d and fixed for microscopy. At least 100 cells were assessed in each experiment, and the experiments were repeated three times. (***A***) Representative DIC images of the control cells (top-left), TC10-depleted cells (top-right), resi-TC10-WT expressing TC10-depleted cells (bottom-left), and resi-TC10-Q75L expressing TC10-depleted cells (bottom-right) are shown. A bar, 15 μm. (***B***) Cells with neurites longer than their cell body lengths were scored as neurite-bearing cells. The results are expressed as the mean plus SE of the percentage of neurite-bearing cells. The symbols indicate the results of a one-way ANOVA followed by Dunnett’s post-hoc test; **p < 0.01.

Next, we investigated NGF-induced formation of mature neurites in TC10 knockdown cells. We used an shRNA expression vector containing the pac gene to allow selection of shRNA-expressing cells with puromycin. In TC10 shRNA-expressing cells, 70% of endogenous TC10 was depleted (Figure S3). PC12 cells transfected with an empty shRNA vector developed neurites within 48 h after NGF addition (Figure 4A, top-left). Depletion of TC10 strongly inhibited neurite outgrowth (Figure 4A, top-right). The proportions of neurite-bearing cells were calculated in control and knockdown cells (Figure 4B). In control cells, the proportion of neurite-bearing cells was 54% in the presence of NGF. Only 11% of TC10 knockdown cells bore neurites in the presence of NGF. Expression of shRNA-resistant wild-type TC10 restored neurite outgrowth. *A significant* decrease in neurite outgrowth by TC10 depletion was also observed in dbcAMP-treated PC12 cells (Figure S4). In conjunction with similar results in TC10-depleted hippocampal neurons [11], these data indicate that TC10 plays a critical role in neurite outgrowth in a broad range of neuronal cells.

To clarify the functions of TC10 in neuritogenesis, we investigated the effects of the expression of a constitutively active or dominant-negative mutant of TC10 in knockdown cells. *A constitutively* active mutant of TC10, TC10-Q75L, did not rescue the decrease in NGF-induced neurite outgrowth by TC10 depletion (Figure 4A, bottom-right; Figure 4B). This suggests that GTP hydrolysis of TC10 near the plasma membrane (Figures 1 and 2) is required for NGF-induced neurite outgrowth. Similarly, constitutive transport of the VSV-*G protein* to the plasma membrane has been reported to be severely impaired by TC10-Q75L expression in 3T3-L1 cells [36]. As well as TC10-Q75L, a dominant-negative mutant of TC10, TC10-T31N, did not rescue the reductions in NGF-induced neuritogenesis by TC10 depletion. This could be explained by the requirement of GTP-TC10 for the recruitment of Exo70 to the vesicles as discussed below (see *Discussion*). The knockdown experiment in Figure 4 was confirmed by the result that the simple overexpression of TC10-Q75L or TC10-T31N in PC12 cells significantly reduced NGF-induced neurite outgrowth (Figure S5). The overexpression of wild-type TC10 slightly promoted neuritogenesis, although not significantly. In HeLa cells, we previously demonstrated the inhibitory effect of TC10-Q75L overexpression on vesicle fusion [20]. By analogy to the study in HeLa cells, we presume that GTP hydrolysis of TC10 promotes neurite outgrowth by accelerating vesicle fusion.

**Figure 5:**
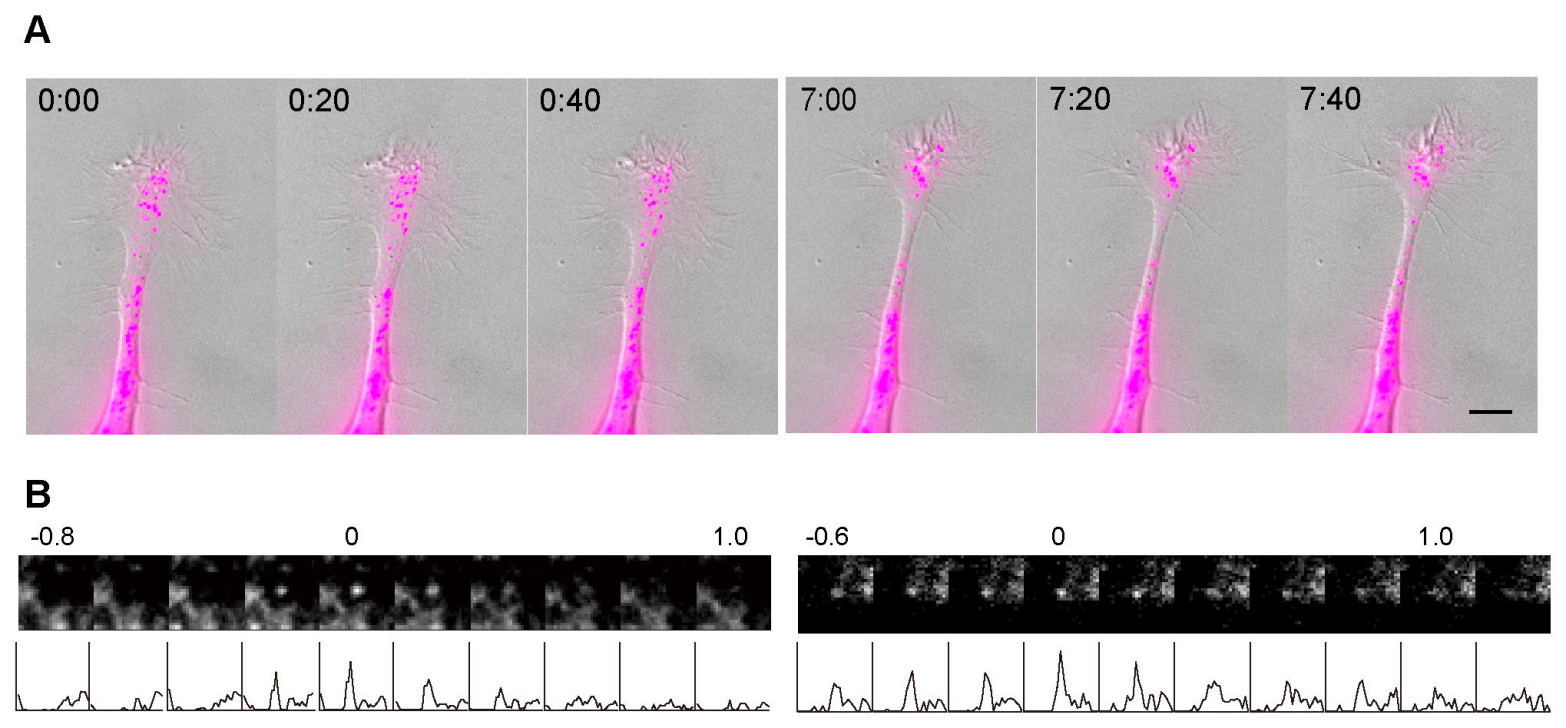
Dynamics of TC10-containing vesicles. (***A***) N1E-115 cells expressing mCherry-TC10 were serum-starved for 6 h and then imaged every 20 sec. Representative mCherry images superimposed with corresponding DIC images at the indicated time points (in min:sec) are shown. A bar, 10 μm. (***B***) The time-lapse TIRF images (3 × 3 μm) showing two examples of fusion events in the cell bodies of PC12 cells expressing mTFP-TC10. Images were obtained at 200 msec intervals. Time point zero was set to the first frame showing the highest intensity of the vesicles. The bottom row in each example shows a plot of fluorescence intensity scanned across the center of a fusing vesicle. This TIRF imaging cannot be executed in growth cones because of their structural fragility.

Dynamics of TC10-containing vesicles supports the presumed role of TC10 in vesicle fusion in neuronal cells. N1E-115 neuroblastoma cells expressing mCherry-TC10 were serum-starved for 6 h to promote growth cone spreading and their growth cones were imaged every 20 sec (Video S3). In growth cones, TC10-positive vesicles were mostly round rather than tubular (Figures 5A and S6A). These TC10 vesicles were obviously enriched in the middle and periphery of central domains, although small vesicles infrequently invaded to nascent flat lamellipodia or filopodia (Figure S6B). The edges of central domains corresponded to transitional zones, where large, clear, and coat-free vesicles, probably PPVs, were transported along microtubules and clustered [1]. During extension, most TC10-positive vesicles repeated outward (0.31 μm/s on average) and inward (0.22 μm/s on average) movements within central domains. We speculate that some TC10-containing vesicles fused to the plasma membrane because these vesicles sometimes disappeared during observation. This presumption is supported by the results of time-lapse TIRF microscopy. Figure 5B showed two examples of fusion events of TC10-positive vesicles in somas of NGF-treated PC12 cells. NGF treatment increased the frequency of fusion events approximately 10-fold in comparison to non-treated cells (unpublished data). Dual-color TIRF microscopy using mCherry-TC10 and L1-GFP in NGF-treated PC12 cells demonstrated that TC10 vesicles fused to the plasma membrane (Figure S7). These results support the idea that TC10 inactivation in close proximity to the plasma membrane plays a role in neurite outgrowth by accelerating vesicle fusion.

### Involvement of Exo70 in TC10-regulated trafficking

**Figure 6:**
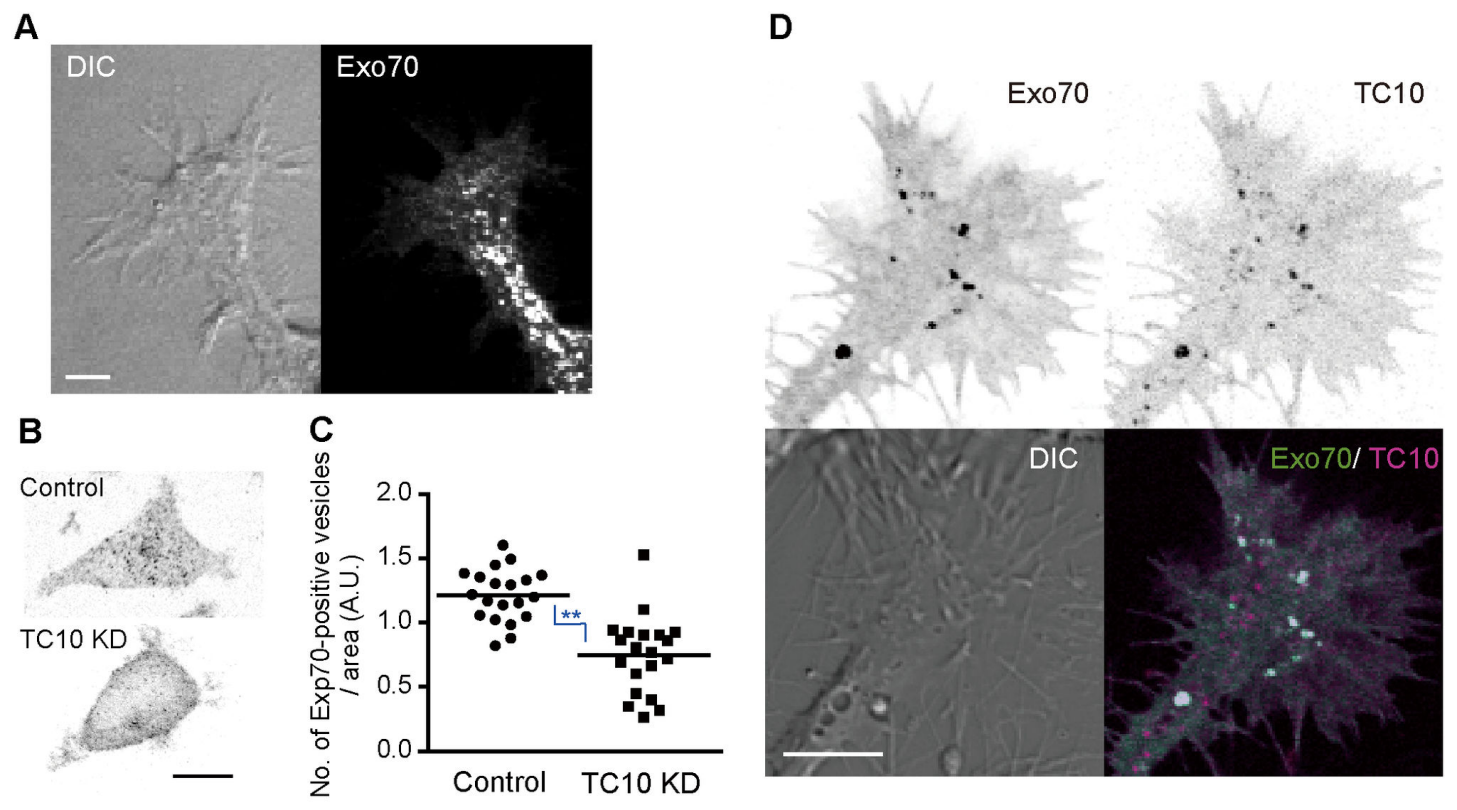
Exo70 distribution in growth cones and effects of TC10 depletion on Exo70-containing vesicles. (***A***) N1E-115 cells expressing EGFP-Exo70 were serum-starved for 6 h, fixed, and confocal images were obtained. Representative DIC and EGFP images of a growth cone and a neurite shaft are shown in a maximum intensity projection mode. A bar, 10 μm. (***B and C***) PC12 cells were transfected with control or TC10-targeted shRNA vector. After selection with puromycin for 2 d, the cells were immunostained with anti-Exo70 antibody and confocal images were obtained. (***B***) A representative Exo70 image of a control or TC10-depleted cell with black and white reversed. A bar, 10 μm. (***C***) A scatter plot depicts the number of vesicles in individual cells in control or TC10 knockdown cells. Bars indicate the median values (n = 20). The blue symbol indicates the result of a Student’s *t* test analysis (**p < 0.01). (***D***) N1E-115 cells expressing EGFP-Exo70 and mCherry-TC10 were serum-starved for 6 h and imaged. Representative images of the subcellular distribution of Exo70 and TC10 in growth cones are shown. Merged images (bottom-right) show remarkable colocalization of Exo70 with TC10 on vesicles. A bar, 10 μm.

The possibility that GTP hydrolysis of TC10 is linked to the acceleration of vesicle fusion in neuritogenesis prompted us to investigate which molecule mediated this function of TC10. Exo70 is a component of the exocyst tethering complex [17] and an effector of TC10 [18]. Studies in Drosophila and mammalian neurons indicate that the exocyst is found at growth cones [37,38]. However, the subcellular localization of Exo70 is still under debate. Several studies reported that Exo70 is associated mainly with the plasma membrane through interaction with PI(4,5)P_2_ [22,23], although localization of Exo70 to endosomes has been described [39,40]. Thus, we examined the distribution of Exo70 in serum-starved N1E-115 cells by confocal microscopy (Figure 6A). In growth cones and neurite shafts, Exo70 clearly localized to vesicular structures, while a certain level of Exo70 was found in the cytoplasm and plasma membrane. This result most closely matches recent observations in HeLa cells [40].

We next examined the effects of TC10 depletion on Exo70 distribution in PC12 cell bodies. This cannot be directly tested in neurite tips because TC10 silencing strongly impairs neuritogenesis. Figures 6B and 6C show that TC10 depletion reduced the number of vesicles containing endogenous Exo70 in PC12 cell bodies by 40% on average. Consistently, about half of TC10-positive vesicles contained Exo70 in growth cones of N1E-115 cells (Figure 6D). *A plausible* explanation is that GTP-TC10 recruits Exo70 to the vesicles through direct binding to a considerable extent. Thus, we suggest that GTP hydrolysis of vesicular TC10 near the plasma membrane leads to the release of Exo70 from TC10. This release may affect the integrity of the exocyst complex containing Exo70 [41]. The role of the exocyst complex in regulating SNARE assembly and vesicle fusion is discussed below (see *Discussion*).

### Characterization of TC10-containing vesicles in neuronal cells

**Figure 7:**
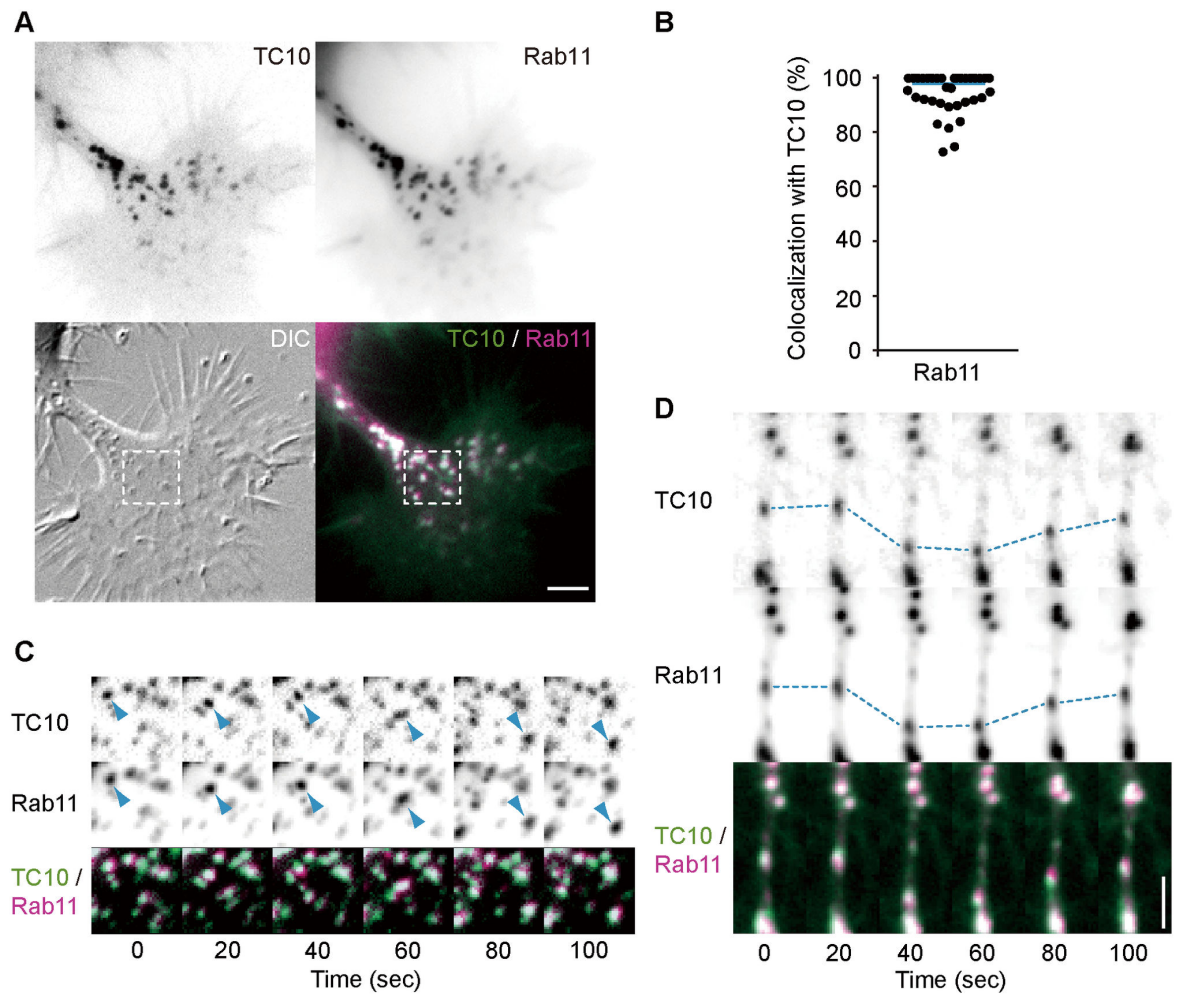
Colocalization of TC10 with Rab11 on vesicles. N1E-115 cells expressing mCherry-Rab11 and mTFP-TC10 were serum-starved for 6 h and imaged every 10 sec. (***A***) A representative image of the subcellular distribution of TC10 and Rab11 in a growth cone. A merged image (bottom-right) shows remarkable colocalization between TC10 and Rab11 on vesicles. A bar, 5 μm. (***B***) A scatter plot depicts the percentage of Rab11-containing vesicles that also have TC10. A blue bar indicates the median value (n = 35). (***C***) mTFP (TC10), mCherry (Rab11), and merged images corresponding to the boxed region in (***A***) at the indicated time points are shown. Blue arrowheads mark the co-migration of TC10 and Rab11 on vesicles. (***D***) Representative images of vesicular localization of TC10 and Rab11 in a neurite shaft at the indicated time points. The movement of one vesicle containing both TC10 and Rab11 is traced with a dotted line. A bar, 5 μm.

To further clarify the role of TC10 in neurite outgrowth, we tried to characterize TC10-positive vesicles in neuronal cells. Membrane addition to neurite tips is thought to be mainly mediated by a recycling pathway [42]. Thus, we examined colocalization of TC10 and Rab11, which plays a central role in regulating a recycling pathway, in growth cones. N1E-115 cells expressing mTFP-TC10 and mCherry-Rab11 were serum-starved for 6 h and their growth cones were imaged every 20 sec. In growth cones, Rab11 localization to vesicles was found to overlap almost completely with that of TC10 (Figure 7A and Video S4). Ninety-eight percent of Rab11 vesicles contained TC10 (Figure 7B). Growth cones of hippocampal neurons had similar levels of colocalization (98.0±0.8%; n = 17) to N1E-115 cells. The colocalization between TC10 and Rab11 in cell bodies and neurite shafts of N1E-115 cells (91.1±3.2%; n = 12) was comparable to that in their growth cones. Furthermore, colocalization of endogenous Rab11 with FLAG-tagged TC10 on vesicle structures in growth cones was confirmed in N1E-115 cells (Figure S8A). We next analyzed the transport behaviours of mTFP-TC10 and mCherry-Rab11 in N1E-115 cells. *A clear* co-migration was observed in growth cones (arrowheads in Figure 7C) and neurite shafts (Figure 7D). Rab11 can bind to another exocyst component, Sec15 [43], and previous work in HeLa cells implies that the exocyst may be recruited onto perinuclear recycling endosomes and then transported to the cell periphery together with Rab11 [21]. On these bases, it might be possible that a measurable amount of exocyst complexes or subcomplexes [44] assemble at the early stage of neurite trafficking and move to growth cones with TC10 and Rab11. TIRF experiments in NGF-treated PC12 cells expressing mTFP-TC10 and dominant-negative mutant of Rab11 (Rab11-S25N) fused to mCherry have shown that the fusion of TC10-positive vesicles is strongly inhibited by Rab11-S25N overexpression (Figure S9). This result supports the functional linkage between Rab11 and TC10 in vesicle trafficking.

**Figure 8:**
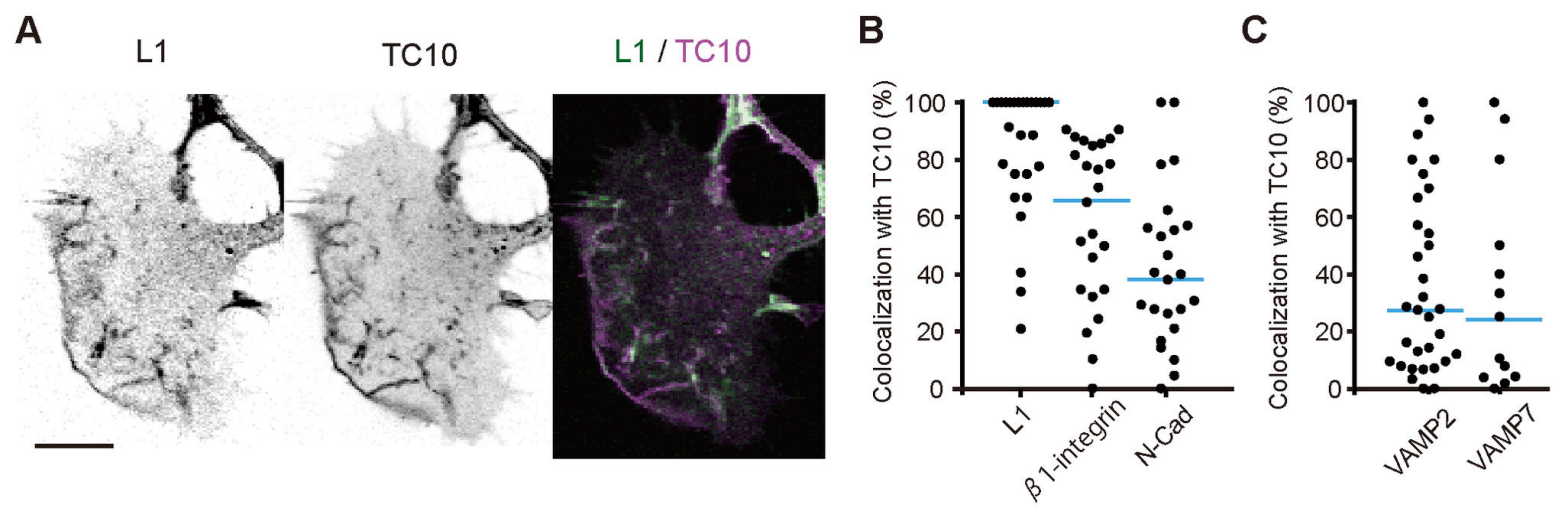
Colocalization of TC10 with L1 on vesicles. (***A and B***) For L1, N1E-115 cells expressing mCherry-TC10 and L1-Venus were serum-starved for 6 h and then imaged. For β1-integrin and N-cadherin, serum-starved N1E-115 cells expressing mCherry-TC10 were examined by immunocytochemistry. (***A***) A representative image of the subcellular distribution of L1 and TC10 in a growth cone. A merged image (right) shows colocalization on vesicles. A bar, 10 μm. (***B***) A scatter plot depicts the percentage of L1-, β1-integrin-, or N-cadherin-containing vesicles that also had TC10 in individual cells. Blue bars indicate the median values. The number of experiments was as follows: 27 L1, 24 β1-integrin, and 24 N-cadherin experiments. (***C***) N1E-115 cells expressing mCherry-TC10 in combination with Venus-VAMP2 or Venus-VAMP7 were serum-starved for 6 h and then fixed for confocal microscopy. A graph showing the distributions of the percentage of VAMP2- or VAMP7-containing vesicles that also contained TC10 in individual cells. Blue bars indicate median values. The number of experiments was as follows: 32 for VAMP2 and 13 for VAMP7.

Furthermore, in N1E-115 cells, we examined an overlap between TC10 and three cell adhesion molecules (CAMs), which have been implicated in neurite outgrowth [45]. L1 and related CAMs are supplied from the soma to the growth cone [46], and concurrently, parts of these CAMs are brought from the rear of the growth cone to its front for reuse [47]. In addition, L1 is supposed to be localized to a fraction of PPVs [11]. In growth cones of N1E-115 cells, most L1-positive vesicles contained TC10 (Figures 8A and 8B). L1/TC10 double-positive vesicles frequently showed an outward movement (unpublished data). In neurite shafts, clear colocalization was also observed, and rapid anterograde and retrograde transport occurred in a large population of L1 vesicles. Colocalization of endogenous L1 with FLAG-tagged TC10 on vesicle structures in growth cones was confirmed in N1E-115 cells (Figure S8B). Sixty-five percent of β1-integrin positive vesicles contained TC10, and 40% of *N-*cadherin vesicles contained TC10 (Figure 8B). This degree of overlap is plausible because Rab11 is implicated in trafficking of β1-integrin and *N-*cadherin during neurite outgrowth or migration [48]. Furthermore, recent work has reported that TC10 and Exo70 have a major role in *N-*cadherin trafficking in prion protein-promoted neurite outgrowth [10].

We next examined the overlap between TC10 and VAMP2 or VAMP7, since several v-SNAREs that include VAMP2 and VAMP7 have a critical role in exocytosis for regulated delivery of membrane in neuritogenesis [27,49,50]. *A quarter* of VAMP2- or VAMP7-positive vesicles were found to contain TC10, although there was considerable variability in the levels of colocalization among individual cells (Figure 8C). This may indicate that separate v-SNAREs are used for TC10-mediated membrane delivery depending on the status of outgrowth.

## Discussion

**Figure 9:**
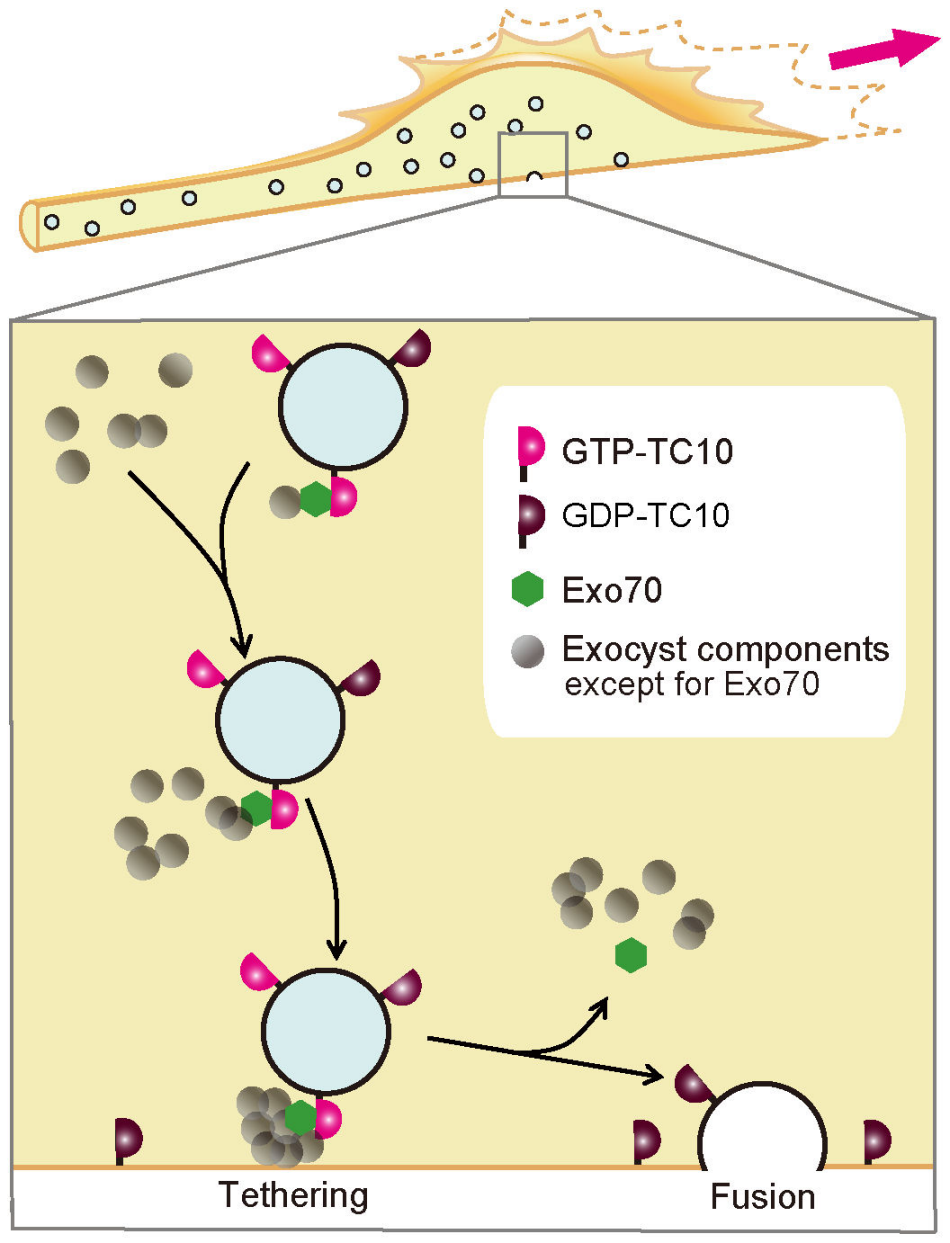
Hypothetical mechanism underlying the neuritogenesis-promoting functions of TC10. The bottom panel exhibits an enlarged view of the boxed region in the top panel, which shows the migration and subsequent fusion of vesicles for membrane expansion in an extending growth cone. In this model, most TC10 on vesicles is GTP-bound and associated with effectors including Exo70. Vesicles containing GTP-TC10 migrate to the plasma membrane. At their destination, vesicle tethering occurs through a full assembly of the exocyst complex containing Exo70 pre-bound to GTP-TC10. Next, GTP hydrolysis of TC10 on the tethered vesicles induces release of Exo70 and exocyst disassembly, which contributes to vesicle fusion.

Here we investigated the molecular mechanism of the role of TC10 in neuritogenesis and propose a model in which the neuritogenesis-promoting functions of TC10 are executed by triggering vesicle fusion through release of Exo70 by GTP hydrolysis (Figure 9). Our key observations are that TC10 activity at the plasma membrane was lower at extending growth cones and that constitutively active TC10 did not rescue the decrease in NGF-induced neurite outgrowth following TC10 depletion. In neuronal cells, TC10 activity at vesicles was higher than that at the plasma membrane, and TC10-positive vesicles were found to fuse to the plasma membrane in NGF-treated PC12 cells. Involvement of Exo70 in TC10-mediated trafficking was supported by the results of TC10 knockdown experiments and colocalization analyses. We emphasize that our observation of lower plasmalemmal TC10 activity at extending growth cones is unexpected because, so far, it has been thought that plasmalemmal TC10 is activated upon stimulation and binds to its effectors there. A recent study has reported that there may be high constitutive activity of TC10 [51]. This is consistent with our idea that the activity of TC10 at vesicles is decreased in proximity to the plasma membrane following stimulation. In addition to its role in neuritogenesis ([11,52] and this study), TC10 is involved in axonal regeneration. In rat motor neurons, axotomy increases the TC10 mRNA level dramatically [52]. Furthermore, reggie/flotilin regulates axon regeneration [53] and reggie-mediated signaling depends partly on TC10- and Exo70-dependent cargo delivery to growth cones [10].

Characterization of TC10 vesicles in neuronal cells has provided evidence for our model. We observed significant colocalization of Rab11 with TC10 on vesicular structures in growth cones, neurite shafts, and cell bodies. This result is in line with the fact that TC10 and Rab11 can bind to the exocyst component, Exo70 or Sec15, respectively [18,43]. Rab11 is the most prominent marker of recycling endosomes (REs) [54,55]. Cells use REs for the delivery of membranes to regions of their surface that are subject to dynamic reorganization, probably through regulated interaction with the exocyst [56]. In addition to TC10, Rab11 plays a role in neuritogenesis. In PC12 cells, adult DRG neurons, and cortical neurons, the expression of constitutively active Rab11 promoted neurite outgrowth [48,57]. In Drosophila, a homozygous mutant Rab11 embryo showed defects in the developing central nervous system, along with disorganization and misrouting of axons [58]. L1 is another molecule that showed clear colocalization with TC10 on vesicles. L1 and related CAMs are supplied from the soma to the growth cone [46]. In addition, L1 is purportedly localized to a fraction of PPVs [11]. These results support the idea that exocytic fusion of Rab11- and L1-containing vesicles to the plasma membrane is a critical component of the functions of TC10 in neurite outgrowth.

This mechanistic view agrees with our observations that a substantial amount of Exo70 resided on vesicles in nerve growth cones. An exocyst complex was found at the tips of growing neurites and growth cones [37,44]. Recently, subcellular fractionation showed that Exo70 was highly enriched in isolated growth cones. However, the question of whether Exo70 is mainly localized to vesicles or the plasma membrane is still being discussed. Several studies reported that Exo70 is associated with the plasma membrane in yeast and mammalian cells [22,23]. On the other hand, in HeLa cells, Exo70 was significantly localized to peripheral and perinuclear structures [21], and half of yeast Exo70 moved to polarized sites via vesicle-dependent routes [42]. The data presented in Figure 6 have supported the latter position, and thus led to our hypothesis that GTP-TC10 binds to Exo70 on vesicles. The presumed role of GTP-TC10 to recruit Exo70 onto vesicles could explain the fact that TC10-T31N did not rescue the decrease in NGF-induced neurite outgrowth by TC10 depletion (Figure 4).

In vesicular fusion, an exocyst complex likely plays active roles in regulating SNARE assembly in addition to simply tethering two lipid bilayers [59,60]. An exocyst can bind to both SNAREs [61] and SM proteins [62], which are dedicated SNARE-binding proteins that control membrane fusion [63]. These kinds of binding can be used to couple tethering to SNARE-mediated membrane fusion for multi-subunit tethering complexes including exocyst [64]. A key aspect to understanding exocyst functions, including SNARE regulation, is to determine the mechanisms underlying its assembly and disassembly, although the link between GTPase binding and exocyst assembly is just being unraveled [44,63]. One interesting possibility is that the abortion of an interaction between GTPases and pairing exocyst components may lead to the disassembly of the exocyst complex. For example, the yeast exocyst component Sec15 is associated with GTP-bound Sec4, a Rab11 ortholog, and assembly of this exocyst depends on this interaction [65]. In mammals, GTP-Ral binds to the exocyst components Sec5 and Exo84 [66,67] and Ral depletion reduces the assembly of the complete exocyst complex [68]. Recently, a TC10 depletion experiment in 293T cells indicated that Exo70 is required for maintaining the integrity of the exocyst complex [41], although the precise mechanism for this is unknown. We think that the present work has provided a clue to unveil the mechanism linking TC10 activity and exocyst integrity, which regulates vesicle fusion.

Coordinated regulation of cytoskeletal reorganization and membrane trafficking is a key issue to understand the mechanisms mediating neurite outgrowth. *A close* coupling between small *G proteins* that are involved in these processes is particularly important. In the present study, this kind of coupling was illustrated by the existence of a Rac1-TC10 pathway ([20] and this study) and the colocalization of TC10 and Rab11 on vesicles (Figure 7). We have proposed that local Rac1 activation at neurite tips may be realized by distinct mechanisms depending on various stimulations, and play a critical role in cytoskeletal reorganization, which leads to neurite outgrowth [24,25,69]. This local Rac1 activation can lead to local TC10 decreases at the neurite tips. Thus, the Rac1-TC10 pathway may be used as a hub in coordinated regulation between cytoskeletal reorganization and membrane trafficking in neuritogenesis. *A functional* coupling between TC10 and Cdc42 is also possible. TC10 can bind to N-WASP [70], which is a well-known effector of Cdc42 [71]. TC10 inactivation at the extending protrusions may increase the amount of free N-WASP, and lead to local increases in binding between active Cdc42 and N-WASP. Thereby, activated *N-*WASP can promote actin reorganization and contribute to neurite outgrowth. Additionally, this study has shown clear colocalization of TC10 and Rab11 on vesicles in neurite shafts and growth cones. An important issue for future study is how the activity and function of these two *G proteins* are linked spatially and temporally. We expect that the analysis using FRET sensors is very useful for clarifying this kind of issue about local couplings among individual signaling molecules.

## Supporting Information

Figure S1 **Spatiotemporal changes of plasmalemmal TC10 activity in stimulated PC12 cells.** (***A***) PC12 cells were cotransfected with pRaichu-TC10/K-RasCT and pERedNLS-Rac1-G12V, stimulated with NGF, and then imaged every 2 min. The mean FRET/CFP ratios averaged over the whole cell are expressed in the same manner as in the legend to Figure 2B. Error bars show the SE (n = 38). (***B***) PC12 cells expressing Raichu-TC10/K-RasCT were treated with 5 μM DPI for 30 min, stimulated with NGF, and then imaged every 2 min. The mean FRET/CFP ratios averaged over the whole cell are expressed in the same manner as in the legend to Figure 2B. Error bars show the SE. The number of experiments for the control condition was eight and for the DPI pretreatments was 88. The blue symbol indicates the result of a Student’s *t* test analysis (**p < 0.01). (***C***) PC12 cells expressing 3HA-TC10 were treated with 1 mM dbcAMP for the indicated periods and then examined by Bos’ pull-down method. Experiments were repeated three times. Average values are shown in the right panel with the SE, as the number of times they increased in comparison to the values of untreated cells.
(TIF)

Figure S2 **Distribution of TC10 activity in hippocampal neurons.** Hippocampal neurons expressing Raichu-TC10/TC10-CT were cultured in a phenol red-free conditioned medium and imaged. (***A***) A DIC image of hippocampal neurons cultured for 1 d after plating. A bar, 10 μm. (***B***) Enlarged CFP and FRET/CFP ratio images which correspond to the boxed region in (***A***). (***C***) A bar graph represents the average of FRET/CFP ratio on the plasma membrane or vesicles with SE. The symbol indicates the result of a Student’s *t* test analysis (**p < 0.01).
(TIF)

Figure S3 **Efficiency of depletion of TC10.** PC12 cells were transfected with an empty or TC10-targeted shRNA vector. After selection with 2 μg/ml puromycin for 2 d, the cells were analyzed by immunoblotting with anti-TC10 or anti-GAPDH antibodies. Experiments were repeated three times. In TC10 shRNA-expressing cells, 70% of endogenous TC10 was depleted on average.
(TIF)

Figure S4 **Effect of TC10 depletion on dbcAMP-induced neurite outgrowth in PC12 cells.** PC12 cells were transfected with an empty or TC10-targeted shRNA vector. After selection with puromycin, the selected cells were cultured with 1 mM dbcAMP for 2 d and fixed for microscopy. At least 100 cells were assessed in each experiment, and the experiments were repeated three times. (***A***) Representative DIC images of the control cells (top) and TC10-depleted cells (bottom) are shown. A bar, 15 μm. (***B***) Cells with neurites the lengths of which were longer than their cell body lengths were scored as neurite-bearing cells. The results are expressed as the mean percentage of neurite-bearing cells with SE. The symbol indicates the result of a Student’s *t* test analysis (**p < 0.01).
(TIF)

Figure S5 **Effect of the expression of TC10-Q75L or TC10-T31N on NGF-induced neurite outgrowth in PC12 cells.** PC12 cells were transfected with pCAGGS-Flag-TC10-WT or its mutants and cultured with 50 ng/ml NGF for 2 d and fixed for microscopy. At least 50 cells were assessed in each experiment, and the experiments were repeated three times. (***A***) Representative DIC images of the control cells (top-left), TC10-WT expressing cells (top-right), TC10-Q75L expressing cells (bottom-left) and TC10-T31N expressing cells (bottom-right) are shown. A bar, 15 μm. (***B***) Cells with neurites the lengths of which were at least twofold longer than their cell body lengths were scored as neurite-bearing cells. The results are expressed as the mean plus SE of the percentage of neurite-bearing cells. The symbols indicate the results of a one-way ANOVA followed by Dunnett’s post-hoc test; **p < 0.01.
(TIF)

Figure S6 **Distribution of TC10-positive vesicles in growth cones.** (***A***) N1E-115 cells expressing mCherry-TC10 were serum-starved for 6 h and then imaged every 20 sec. Representative mCherry images with black and white reversed and corresponding DIC images at the indicated time points (in min:sec) are shown. A bar, 5 μm. (***B***) N1E-115 cells expressing mCherry-TC10 were serum-starved for 6 h and imaged. Representative images of infrequent invasion events of TC10 vesicles to nascent flat lemellipodia or filopodia are shown with black and white reversed. Blue arrowheads mark invasion events. A bar, 5 μm.
(TIF)

Figure S7 **Fusion of TC10-positive vesicles to the plasma membrane.** The time-lapse dual-color TIRF images (top: 5 × 5 μm, bottom: 2 × 2 μm) showing two examples of fusion events in the cell bodies of PC12 cells expressing mCherry-TC10 (top) and L1-GFP (bottom). Images were obtained at 200 msec intervals. Time point zero was set to the first frame showing the highest intensity of the vesicles. Fusion points are indicated by blue arrowheads.
(TIF)

Figure S8 **Colocalization of TC10 with Rab11 and L1 on vesicles.** (***A***) N1E-115 cells transfected with pCMV-FLAG-TC10 were serum-starved for 6 h and examined by immunocytochemistry. The expression level of FLAG-tagged TC10 in neuronal cells was estimated to be almost comparable to that of endogenous TC10 in our experimental condition (unpublished data). Representative images of the distribution of FLAG-tagged TC10 (left) and endogenous Rab11 (center) in growth cones. Merged images (right) show remarkable colocalization between TC10 and Rab11 on vesicles. A bar, 5 μm. (***B***) N1E-115 cells transfected with pCMV-FLAG-TC10 were serum-starved for 6 h and examined by immunocytochemistry. Representative images of the distribution of FLAG-tagged TC10 (left) and endogenous L1 (center) in growth cones. Merged images (right) show remarkable colocalization between TC10 and L1 on vesicles. A bar, 5 μm.
(TIF)

Figure S9 **Effect of Rab11-S25N expression on fusion of TC10 vesicles.** PC12 cells expressing mTFP-TC10 only or mTFP-TC10 and mCherry-Rab11-S25N were treated with 50 ng/ml of NGF and examined by TIRF microscopy. (***A***) Representative frames of mTFP-TC10 images are shown for control (top) and Rab11-S25N co-expressed (bottom) cells. Bars, 10 μm. (***B***) A bar graph represents the average plus SE of fusion events per 100 μm^2^ during a 6 min observation. The symbol indicates the result of a Student’s *t* test analysis (*p < 0.05).
(TIF)

Video S1 **Spatiotemporal changes in TC10 activity at the plasma membrane of extending neurites.** PC12 cells expressing Raichu-TC10/K-RasCT were treated with 50 ng/ml NGF for 24 h and then imaged every 2 min for 1 h. The numbers indicate the elapsed time (in min). A bar, 5 μm.
(MP4)

Video S2 **Spatiotemporal changes in plasmalemmal TC10 activity following NGF stimulation in PC12 cells.** PC12 cells expressing Raichu-TC10/K-RasCT were starved for 30 min and then stimulated with 50 ng/ml NGF. Images were obtained every 2 min for 20 min after NGF addition. The numbers indicate the elapsed time (in min). A bar, 10 μm.
(MP4)

Video S3 **Dynamics of TC10-containing vesicles in growth cones and neurite shafts.** N1E-115 cells expressing mCherry-TC10 were serum-starved for 6 h and then imaged every 20 sec. Representative mCherry images superimposed with corresponding DIC images are shown. The numbers indicate the elapsed time (in min:sec). A bar, 10 μm.
(MP4)

Video S4 **Colocalization of TC10 with Rab11 on vesicles.** N1E-115 cells expressing mCherry-Rab11 and mTFP-TC10 were serum-starved for 6 h and imaged every 10 sec. Merged image of the subcellular distribution of TC10 (cyan) and Rab11 (magenta) in a growth cone are shown. The numbers indicate the elapsed time (in min). A bar, 5 μm.
(MP4)
