# Supplementary figures and images for: GTP Hydrolysis of TC10 Promotes Neurite Outgrowth through Exocytic Fusion of Rab11- and L1-Containing Vesicles by Releasing Exocyst Component Exo70
Source: PLoS One. 2013 Nov 4;8(11):e79689. doi: 10.1371/journal.pone.0079689 (PMC3817099; doi:10.1371/journal.pone.0079689)

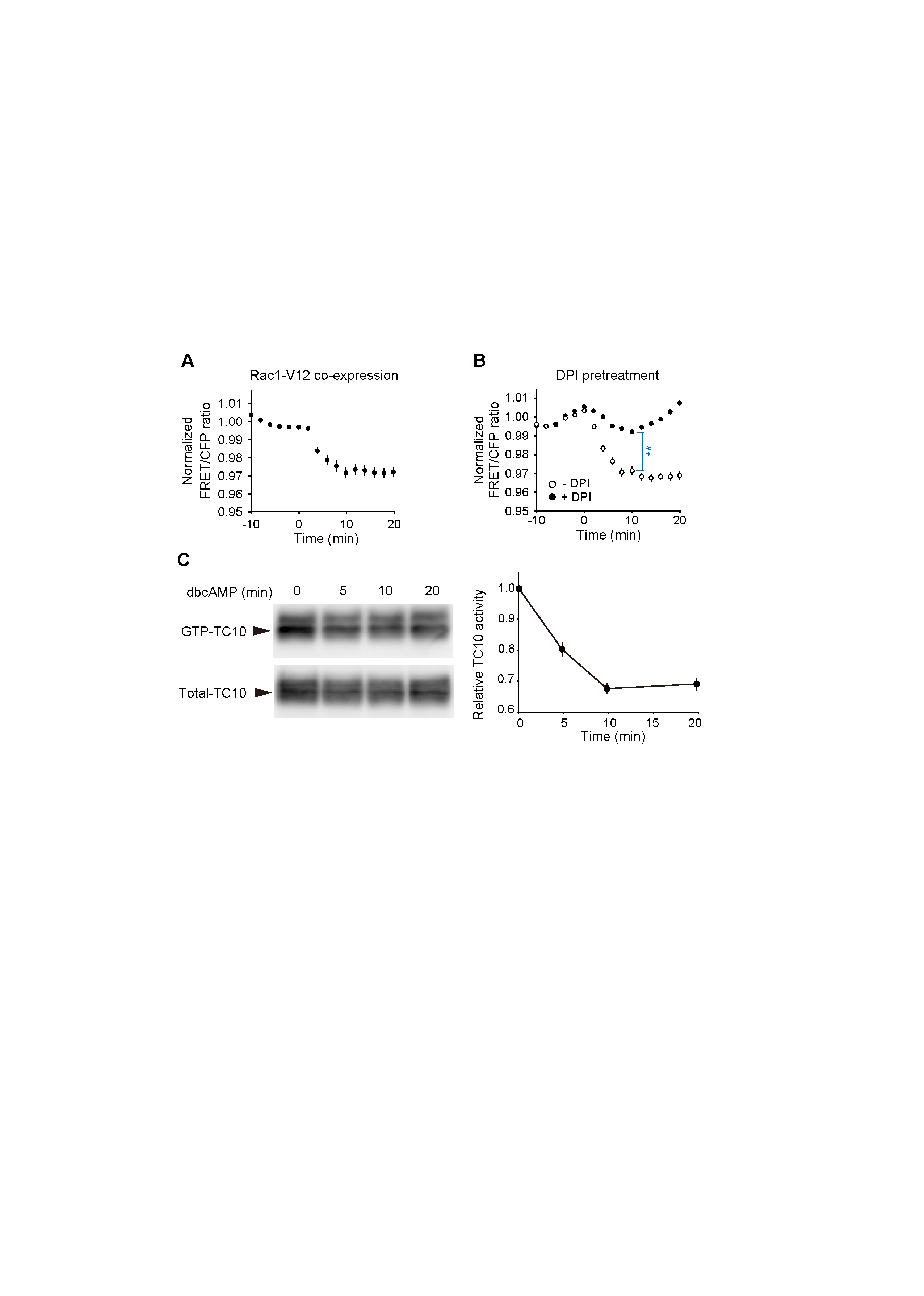

Supplement: Figure S1 — Spatiotemporal changes of plasmalemmal TC10 activity in stimulated PC12 cells. (A) PC12 cells were cotransfected with pRaichu-TC10/K-RasCT and pERedNLS-Rac1-G12V, stimulated with NGF, and then imaged every 2 min. The mean FRET/CFP ratios averaged over the whole cell are expressed in the same manner as in the legend to Figure 2B. Error bars show the SE (n = 38). (B) PC12 cells expressing Raichu-TC10/K-RasCT were treated with 5 μM DPI for 30 min, stimulated with NGF, and then imaged every 2 min. The mean FRET/CFP ratios averaged over the whole cell are expressed in the same manner as in the legend to Figure 2B. Error bars show the SE. The number of experiments for the control condition was eight and for the DPI pretreatments was 88. The blue symbol indicates the result of a Student’s t test analysis (**p < 0.01). (C) PC12 cells expressing 3HA-TC10 were treated with 1 mM dbcAMP for the indicated periods and then examined by Bos’ pull-down method. Experiments were repeated three times. Average values are shown in the right panel with the SE, as the number of times they increased in comparison to the values of untreated cells. (TIF) [file pone.0079689.s001.tif]

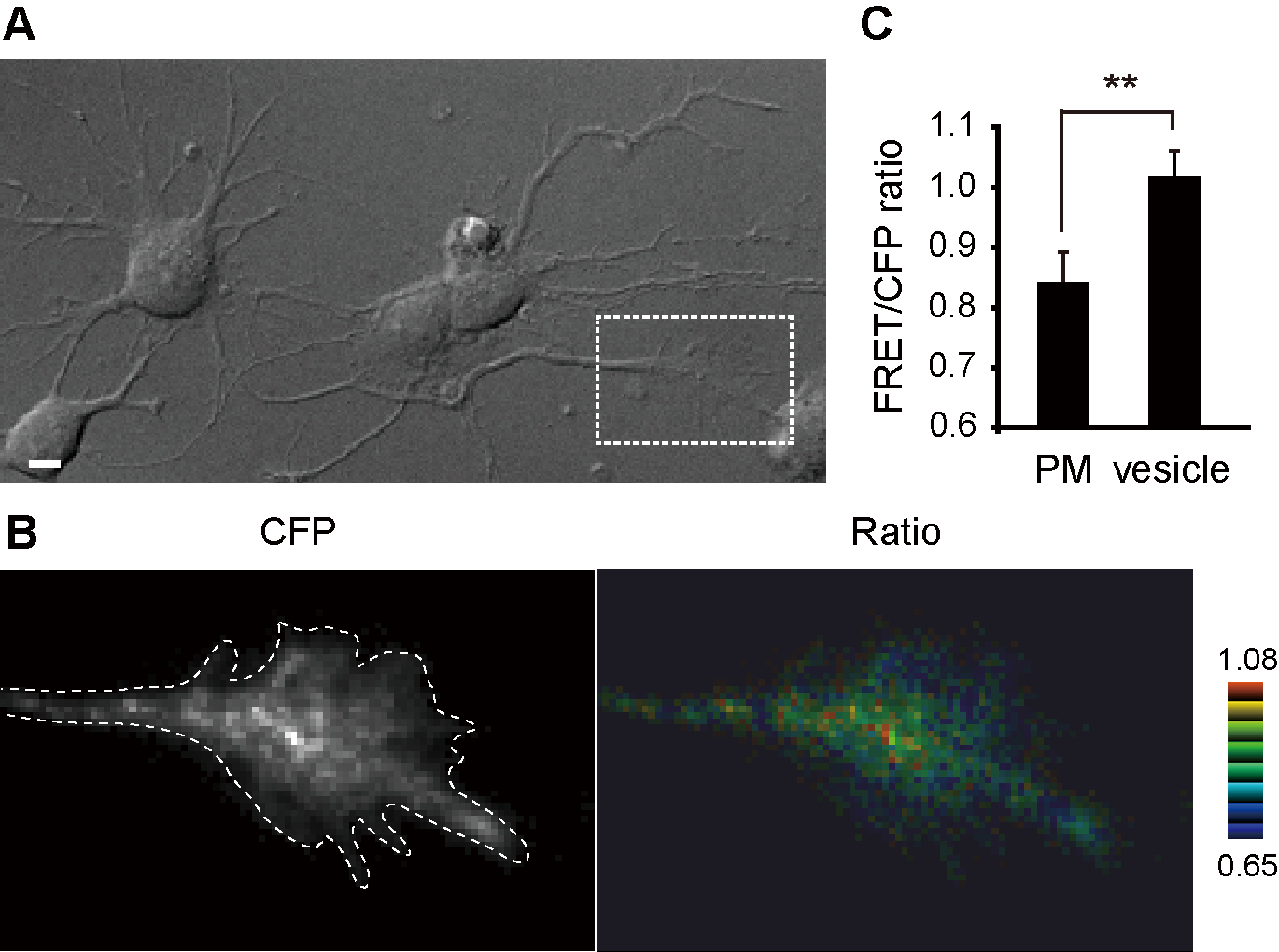

Supplement: Figure S2 — Distribution of TC10 activity in hippocampal neurons. Hippocampal neurons expressing Raichu-TC10/TC10-CT were cultured in a phenol red-free conditioned medium and imaged. (A) A DIC image of hippocampal neurons cultured for 1 d after plating. A bar, 10 μm. (B) Enlarged CFP and FRET/CFP ratio images which correspond to the boxed region in (A). (C) A bar graph represents the average of FRET/CFP ratio on the plasma membrane or vesicles with SE. The symbol indicates the result of a Student’s t test analysis (**p < 0.01). (TIF) [file pone.0079689.s002.tif]

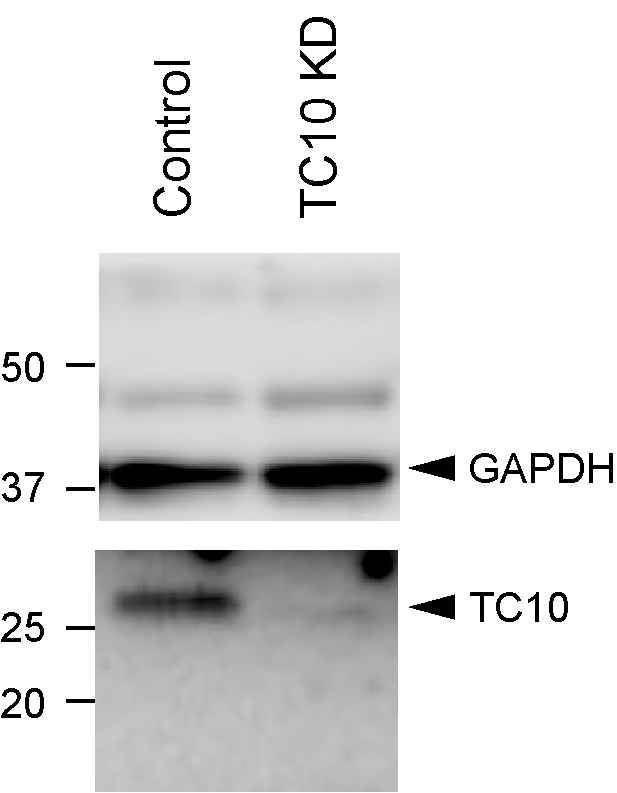

Supplement: Figure S3 — Efficiency of depletion of TC10. PC12 cells were transfected with an empty or TC10-targeted shRNA vector. After selection with 2 μg/ml puromycin for 2 d, the cells were analyzed by immunoblotting with anti-TC10 or anti-GAPDH antibodies. Experiments were repeated three times. In TC10 shRNA-expressing cells, 70% of endogenous TC10 was depleted on average. (TIF) [file pone.0079689.s003.tif]

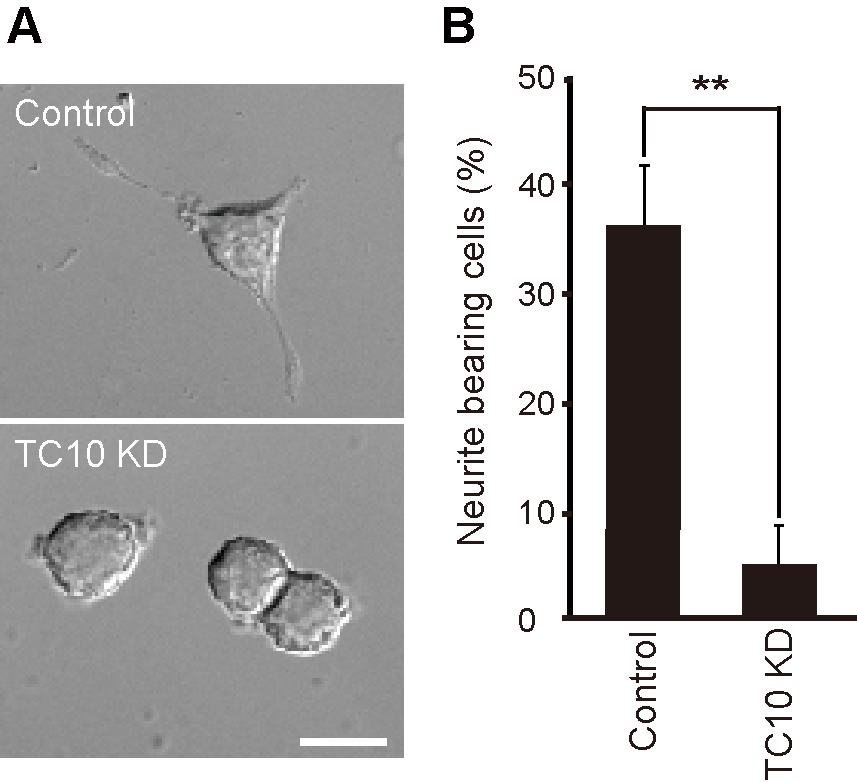

Supplement: Figure S4 — Effect of TC10 depletion on dbcAMP-induced neurite outgrowth in PC12 cells. PC12 cells were transfected with an empty or TC10-targeted shRNA vector. After selection with puromycin, the selected cells were cultured with 1 mM dbcAMP for 2 d and fixed for microscopy. At least 100 cells were assessed in each experiment, and the experiments were repeated three times. (A) Representative DIC images of the control cells (top) and TC10-depleted cells (bottom) are shown. A bar, 15 μm. (B) Cells with neurites the lengths of which were longer than their cell body lengths were scored as neurite-bearing cells. The results are expressed as the mean percentage of neurite-bearing cells with SE. The symbol indicates the result of a Student’s t test analysis (**p < 0.01). (TIF) [file pone.0079689.s004.tif]

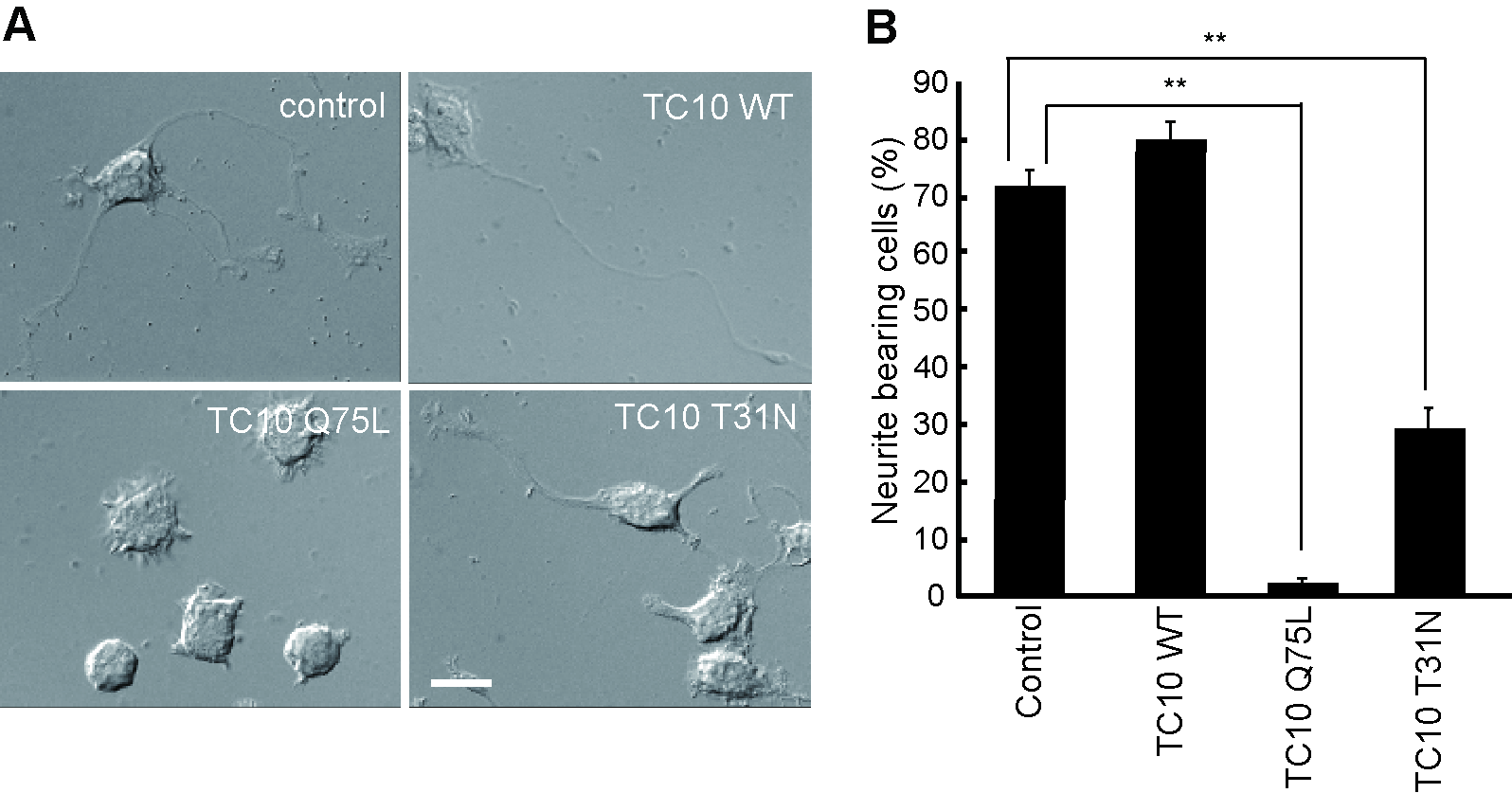

Supplement: Figure S5 — Effect of the expression of TC10-Q75L or TC10-T31N on NGF-induced neurite outgrowth in PC12 cells. PC12 cells were transfected with pCAGGS-Flag-TC10-WT or its mutants and cultured with 50 ng/ml NGF for 2 d and fixed for microscopy. At least 50 cells were assessed in each experiment, and the experiments were repeated three times. (A) Representative DIC images of the control cells (top-left), TC10-WT expressing cells (top-right), TC10-Q75L expressing cells (bottom-left) and TC10-T31N expressing cells (bottom-right) are shown. A bar, 15 μm. (B) Cells with neurites the lengths of which were at least twofold longer than their cell body lengths were scored as neurite-bearing cells. The results are expressed as the mean plus SE of the percentage of neurite-bearing cells. The symbols indicate the results of a one-way ANOVA followed by Dunnett’s post-hoc test; **p < 0.01. (TIF) [file pone.0079689.s005.tif]

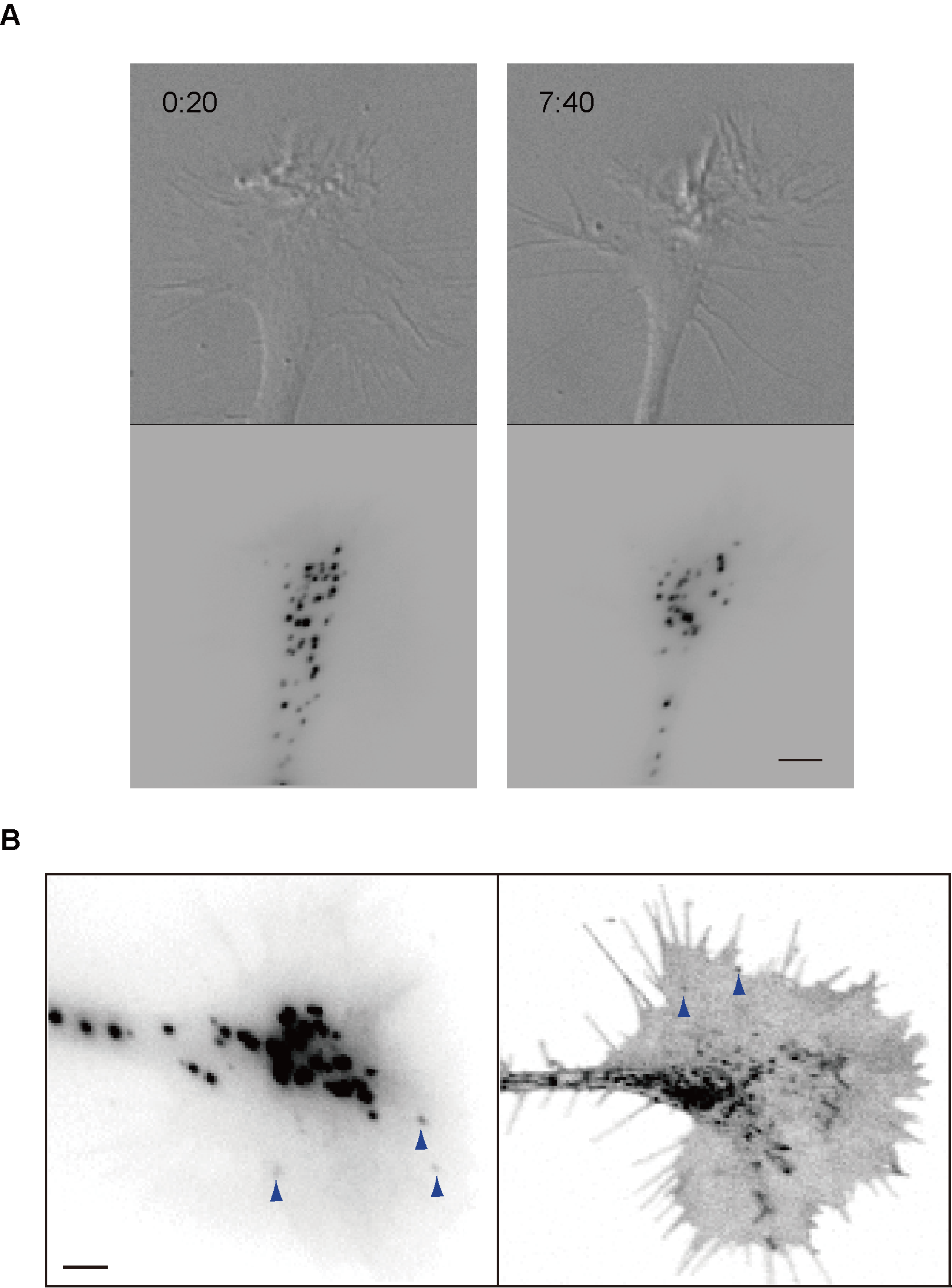

Supplement: Figure S6 — Distribution of TC10-positive vesicles in growth cones. (A) N1E-115 cells expressing mCherry-TC10 were serum-starved for 6 h and then imaged every 20 sec. Representative mCherry images with black and white reversed and corresponding DIC images at the indicated time points (in min:sec) are shown. A bar, 5 μm. (B) N1E-115 cells expressing mCherry-TC10 were serum-starved for 6 h and imaged. Representative images of infrequent invasion events of TC10 vesicles to nascent flat lemellipodia or filopodia are shown with black and white reversed. Blue arrowheads mark invasion events. A bar, 5 μm. (TIF) [file pone.0079689.s006.tif]

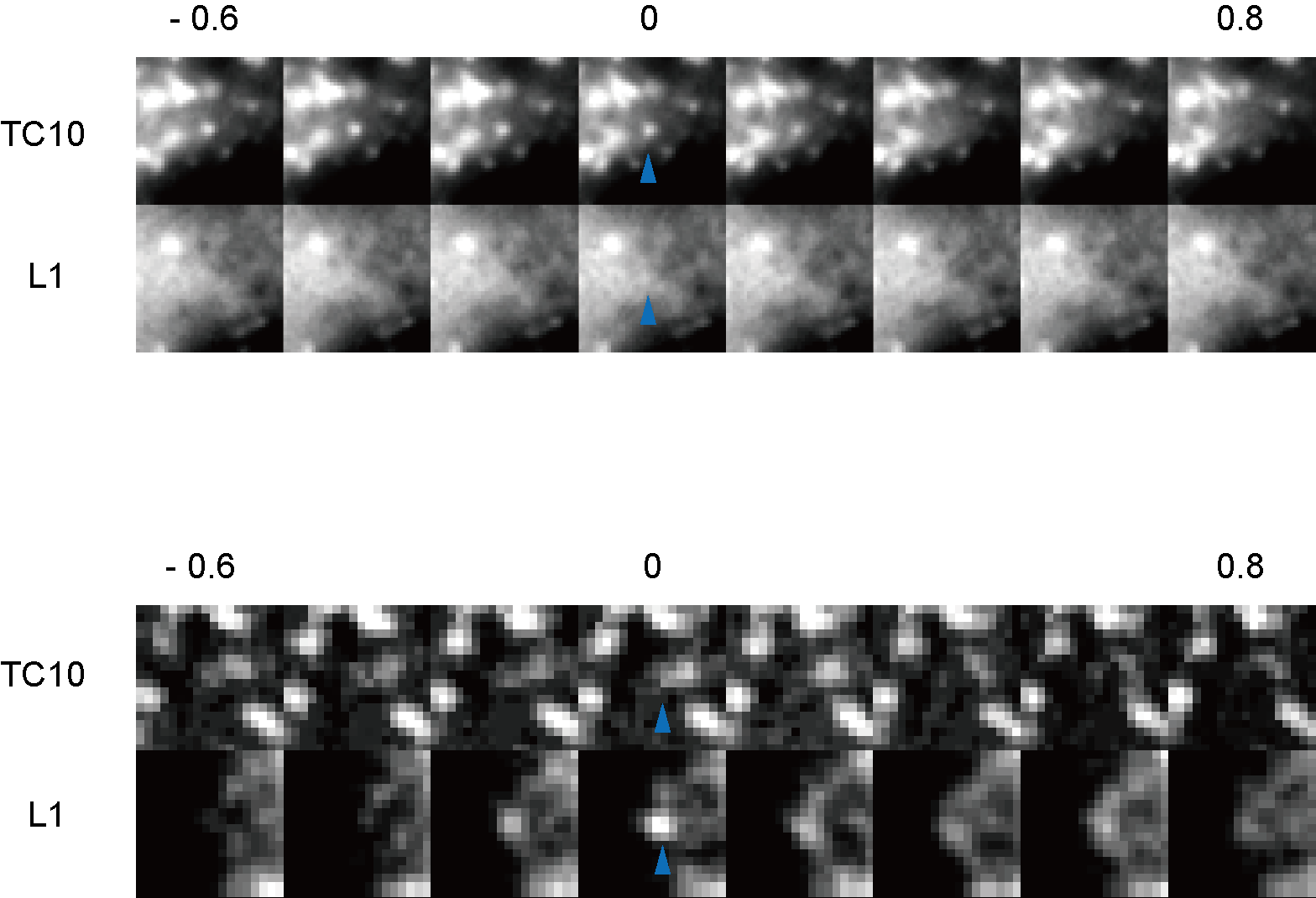

Supplement: Figure S7 — Fusion of TC10-positive vesicles to the plasma membrane. The time-lapse dual-color TIRF images (top: 5 × 5 μm, bottom: 2 × 2 μm) showing two examples of fusion events in the cell bodies of PC12 cells expressing mCherry-TC10 (top) and L1-GFP (bottom). Images were obtained at 200 msec intervals. Time point zero was set to the first frame showing the highest intensity of the vesicles. Fusion points are indicated by blue arrowheads. (TIF) [file pone.0079689.s007.tif]

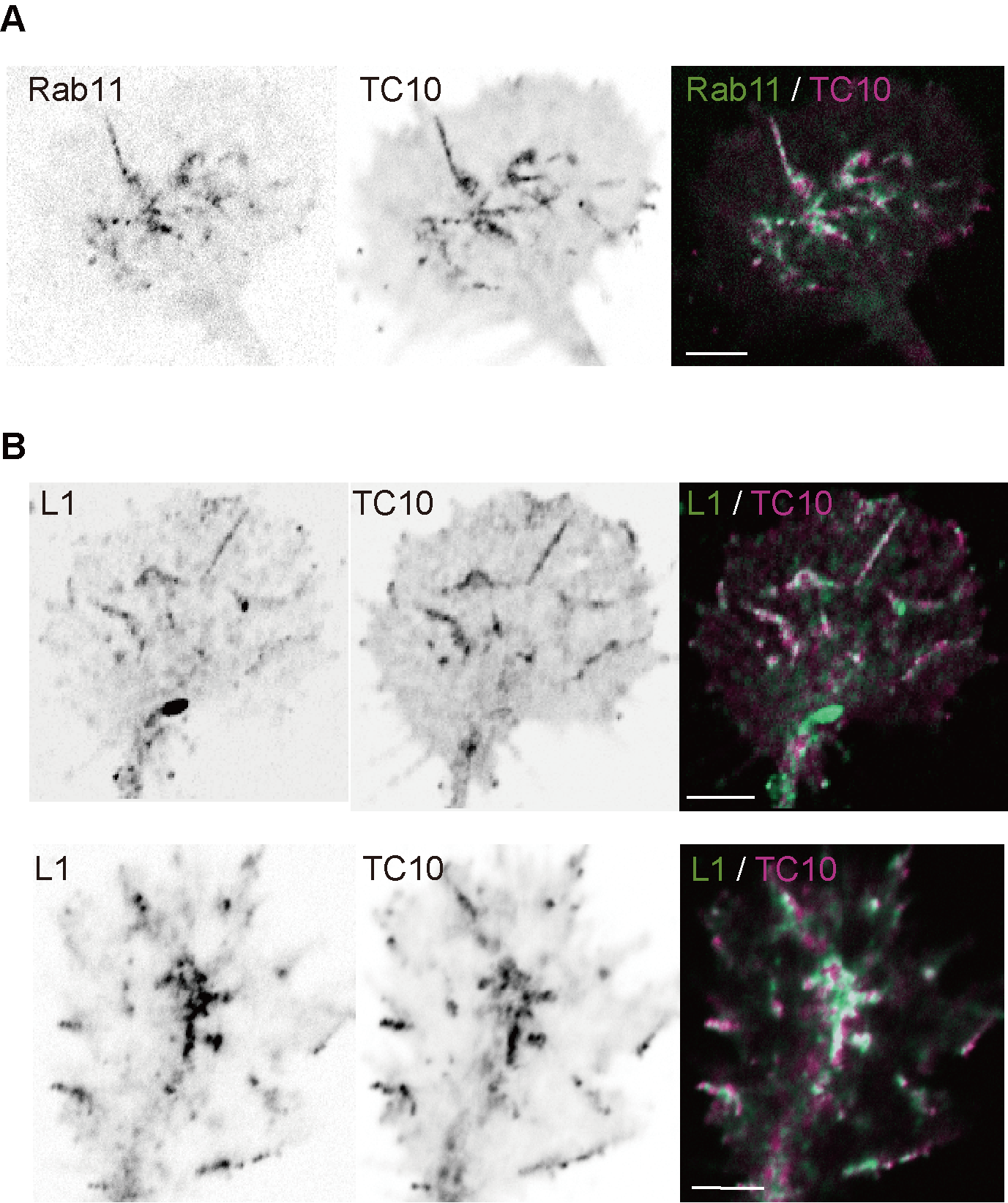

Supplement: Figure S8 — Colocalization of TC10 with Rab11 and L1 on vesicles. (A) N1E-115 cells transfected with pCMV-FLAG-TC10 were serum-starved for 6 h and examined by immunocytochemistry. The expression level of FLAG-tagged TC10 in neuronal cells was estimated to be almost comparable to that of endogenous TC10 in our experimental condition (unpublished data). Representative images of the distribution of FLAG-tagged TC10 (left) and endogenous Rab11 (center) in growth cones. Merged images (right) show remarkable colocalization between TC10 and Rab11 on vesicles. A bar, 5 μm. (B) N1E-115 cells transfected with pCMV-FLAG-TC10 were serum-starved for 6 h and examined by immunocytochemistry. Representative images of the distribution of FLAG-tagged TC10 (left) and endogenous L1 (center) in growth cones. Merged images (right) show remarkable colocalization between TC10 and L1 on vesicles. A bar, 5 μm. (TIF) [file pone.0079689.s008.tif]

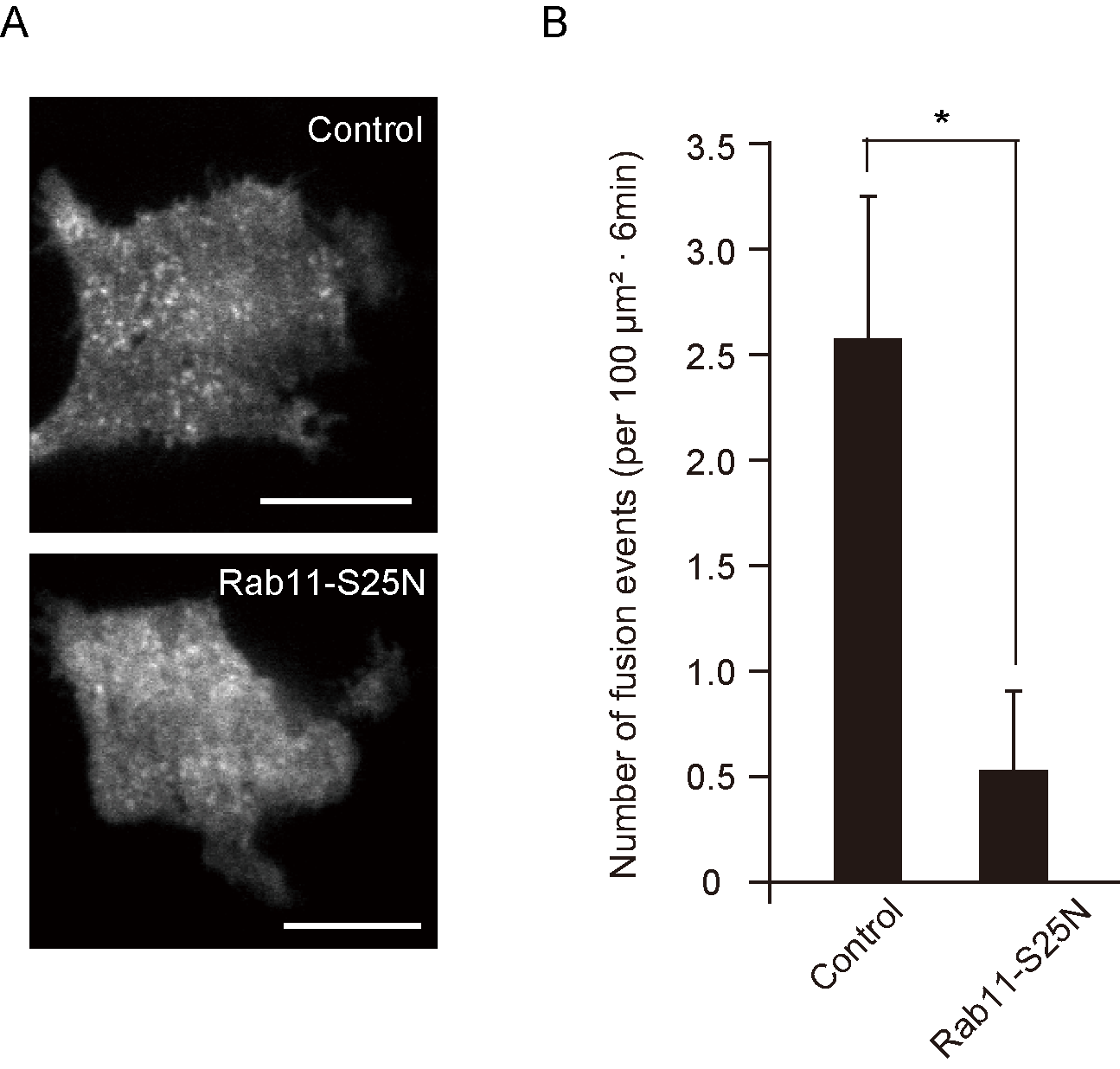

Supplement: Figure S9 — Effect of Rab11-S25N expression on fusion of TC10 vesicles. PC12 cells expressing mTFP-TC10 only or mTFP-TC10 and mCherry-Rab11-S25N were treated with 50 ng/ml of NGF and examined by TIRF microscopy. (A) Representative frames of mTFP-TC10 images are shown for control (top) and Rab11-S25N co-expressed (bottom) cells. Bars, 10 μm. (B) A bar graph represents the average plus SE of fusion events per 100 μm2 during a 6 min observation. The symbol indicates the result of a Student’s t test analysis (*p < 0.05). (TIF) [file pone.0079689.s009.tif]
